# EWSR1 prevents the induction of aneuploidy through direct regulation of Aurora B

**DOI:** 10.3389/fcell.2023.987153

**Published:** 2023-02-15

**Authors:** Haeyoung Kim, Hyewon Park, Evan T. Schulz, Yoshiaki Azuma, Mizuki Azuma

**Affiliations:** Molecular Biosciences, University of Kansas, Lawrence, KS, United States

**Keywords:** mitosis, Aurora B, sarcoma, aneuploidy, chromosome mis-segregation, auxin inducible degron (AID) system

## Abstract

*EWSR1 (Ewing sarcoma breakpoint region 1)* was originally identified as a part of an aberrant *EWSR1/FLI1* fusion gene in Ewing sarcoma, the second most common pediatric bone cancer. Due to formation of the *EWSR1/FLI1* fusion gene in the tumor genome, the cell loses one wild type *EWSR1* allele. Our previous study demonstrated that the loss of *ewsr1a* (homologue of human *EWSR1*) in zebrafish leads to the high incidence of mitotic dysfunction, of aneuploidy, and of tumorigenesis in the *tp53* mutant background. To dissect the molecular function of EWSR1, we successfully established a stable DLD-1 cell line that enables a conditional knockdown of EWSR1 using an Auxin Inducible Degron (AID) system. When both *EWSR1* genes of DLD-1 cell were tagged with *mini-AID* at its 5′-end using a CRISPR/Cas9 system, treatment of the (*AID-EWSR1/AID-EWSR1*) DLD-1 cells with a plant-based Auxin (AUX) led to the significant levels of degradation of AID-EWSR1 proteins. During anaphase, the *EWSR1* knockdown (AUX+) cells displayed higher incidence of lagging chromosomes compared to the control (AUX-) cells. This defect was proceeded by a lower incidence of the localization of Aurora B at inner centromeres, and by a higher incidence of the protein at Kinetochore proximal centromere compared to the control cells during pro/metaphase. Despite these defects, the EWSR1 knockdown cells did not undergo mitotic arrest, suggesting that the cell lacks the error correction mechanism. Significantly, the EWSR1 knockdown (AUX+) cells induced higher incidence of aneuploidy compared to the control (AUX-) cells. Since our previous study demonstrated that EWSR1 interacts with the key mitotic kinase, Aurora B, we generated replacement lines of *EWSR1-mCherry* and *EWSR1:R565A-mCherry* (a mutant that has low affinity for Aurora B) in the (*AID-EWSR1/AID-EWSR1*) DLD-1 cells. The EWSR1-mCherry rescued the high incidence of aneuploidy of EWSR1 knockdown cells, whereas EWSR1-mCherry:R565A failed to rescue the phenotype. Together, we demonstrate that EWSR1 prevents the induction of lagging chromosomes, and of aneuploidy through the interaction with Aurora B.

## Introduction

The *Ewing sarcoma region 1* gene (*EWSR1*) was originally identified in the pediatric bone cancer, Ewing sarcoma, as a part of an aberrant fusion gene with *FLI1* (Delattre et al., 1992). Subsequent studies showed that *EWSR1* is fused to various types of transcription factors in multiple sarcomas (e.g., *EWSR1-WT1*; desmoplastic small round cell tumor, *EWSR1-ATF1*;clear cell sarcoma, *EWSR1-CHOP*; myxoid liposarcoma, *EWSR1-NR4A3*; extraskeletal myxoid chondrosarcoma) (Lee et al., 1997; Panagopoulos et al., 2002; Matsui et al., 2006; Filion et al., 2009). The EWSR1 has various activities in multiple biological phenomena. For example, EWSR1 interacts with the subunits of TFIID and RNA PolII, and regulates transcription of Oct4 and Brn3a (Zhang et al., 1998; Gascoyne et al., 2004; Lee et al., 2005). The EWSR1 regulates splicing through interaction with splicing factor SF1 as well as small nuclear ribonucleoprotein-specific protein U1C that is required for the early spliceosome formation (Zhang et al., 1998; Knoop and Baker, 2000). Other reports demonstrated that EWSR1 promotes homologous recombination by suppressing R-loop formation (Gorthi et al., 2018). Multiple phenotypes were also reported using animal models with genetically ablated *EWSR1.* The knockout mice for EWSR1 displayed impaired differentiation of pre-B lymphocytes, defects in meiosis of sperm, and reduction of mitochondria through degradation of PGC1a and of mitochondria function in brown fat and skeletal muscles of *EWSR1*-deficient mice (Li et al., 2007; Park et al., 2015). Our laboratory discovered that the zebrafish *ewsr1a* (homologue of human *EWSR1*) mutant displayed impaired differentiation of chondrocytes due to the compromised EWSR1-Sox9 dependent transcription (Merkes et al., 2015). In addition, the zebrafish *ewsr1a* (homologue of human *EWSR1*) mutant displays increased incidence of mitotic dysfunction, aneuploidy, and an increased incidence of tumorigenesis in a *tp53* mutation background (Azuma et al., 2007; Park et al., 2016). The study suggested that the loss of EWSR1 may be a part of the molecular pathogenesis of EWSR1-expressing sarcomas because its tumor cells lack one *EWSR1* allele due to the formation of the fusion gene. One important question that arose from this study is whether loss of EWSR1 leads to aneuploidy after 1 cell cycle, or if an aneuploid cell population expands by undergoing a selection process. Therefore, this study aimed to elucidate whether loss of EWSR1 in 1 cell cycle is sufficient to induce aneuploidy, and to characterize the mechanism of the induction of aneuploidy.

A major cause of the induction of aneuploidy is chromosomal mis-segregation derived from defects in microtubule-kinetochore attachment, in altered spindle microtubule dynamics, or in cohesion and condensation of chromosomes during mitosis (Lengauer et al., 1997; Coschi et al., 2010; Solomon et al., 2011). One form of chromosomal mis-segregation is due to chromosome bridges where a stretched DNA strand connects two segregating chromosomes during anaphase. Chromosome bridges are often derived from dicentric chromosomes, from chromosomes with aberrant condensation/cohesion, or from tangled DNA originating from replication stresses (Hauf et al., 2001; Hetzer, 2010). Other form of chromosomal mis-segregation is derived from lagging chromosomes that are “left behind chromosomes” between segregating chromosomes during anaphase. The lagging chromosomes are often derived from merotelic attachment, an occasion of both microtubules in which nucleated from opposite centrioles attaching to the same kinetochore of one chromosome (Cimini et al., 2001b; Funk et al., 2016). Lagging chromosomes result in aneuploidy because they are unevenly distributed in two daughter cells, resulting in either gain or loss of the chromosomes (Cimini et al., 2001a). One important regulator of faithful chromosomal segregation is the Aurora B kinase (Aurora B), a component of chromosome passenger complex (CPC) that includes three additional components, INCENP, Borealin and Survivin (Adams et al., 2000; Kaitna et al., 2000). The Aurora B mainly localizes at inner centromeres and kinetochores during pro/metaphase, and later, it is relocated to the spindle midzone (an structure with anti-parallel microtubules formed between segregating chromosomes) during anaphase (Gruneberg et al., 2004; Cimini et al., 2006; Hümmer and Mayer, 2009; Hadders and Lens, 2022). During pro/metaphase, microtubules are nucleated from the centrosomes located at the opposite ends of the cell, and undergo dynamic instability until they attach to the each side of a kinetochore (amphitelic attachment). Aurora B prevents the induction of lagging chromosomes by destabilizing the site of merotelic attachment by phosphorylating Hec1, MCAK and Kif2b. Aurora B also activates the spindle assembly checkpoint (SAC) by enhancing the localization of SAC proteins Mps1 and BubR1 to maintain checkpoint signaling (Gurden et al., 2018) and it also activates the tension checkpoint (also known as error correction) by phosphorylating outer kinetochore proteins such as KMN network (Knl1, Mis12 and Ndc80) (Welburn et al., 2010; DeLuca et al., 2011). In general, when a cell undergoes defects during mitosis, the cell arrests at in prometaphase during the error correction process (Gorbsky, 1997). This coordination prevents the cell from dividing and to mis-segregating its chromosomes without repairing its errors.

Our previous study demonstrated that the 565th Arg of EWSR1 that is located in its RGG (Arg-Gly-Gly) 3 domain was the critical amino acid required for the interaction with Aurora B, and it is essential for the localization of Aurora B at the spindle midzone during anaphase (Park et al., 2014). Our subsequent study demonstrated that the *ewsr1a/ewsr1a* homozygous zebrafish mutant induces mitotic dysfunction, aneuploidy and both *ewsr1a/wt* heterozygous and *ewsr1a/ewsr1a* homozygous zebrafish have increased rates of tumorigenesis in a *tp53/wt* mutant background (Park et al., 2016). In this study, we asked if the function of EWSR1 in preventing aneuploidy is conserved in human cells. We aimed to address whether the loss of EWSR1 induces aneuploidy after 1 cell cycle, or if aneuploidy is enriched as a result of a selection process after multiple cell divisions. To address these questions, the Auxin Inducible Degron system (AID) was employed in this study as an excellent means to degrade our target protein conditionally. In normal amphitelic attachment, microtubules from one centrosome attach to the kinetochore on one chromatid and microtubules from the other centrosome attach to the the kinetochore on the other chromatid. The two sister chromatids are, thus, pulled in opposite directions by microtubules facilitating correct segregation of sister chromatids in anaphase. Specifically, the system requires the tagging of the protein with a plant specific ubiquitination sequence, *miniAID* (mAID), within cells expressing OsTIR1 that is a plant specific E3 ligase. Therefore, administration of a plant phytohormone, Auxin (AUX), results in the conditional degradation of the protein (Natsume et al., 2016). In this study, using CRISPR/Cas9 system, we successfully established a stable line that is homozygous for *mAID* tagged *EWSR1* alleles (*AID-EWSR1/AID-EWSR1*) at a homozygous level in OsTIR1 expressing DLD-1 cells (Hassebroek et al., 2020). The DLD-1 (a colorectal cancer) cell line was utilized in this study because it carries a near-diploid karyotype, and has been utilized as a model cell line to study the induction of aneuploidy (Lengauer et al., 1997; Nicholson et al., 2015). Here, we show that the knockdown of EWSR1 for 1 cell cycle is sufficient to induce lagging chromosomes and aneuploidy without inducing mitotic arrest. We demonstrate that impairment of the EWSR1-Aurora B interaction overrides the error correction mechanism that prevents the induction of aneuploidy.

## Methods

### Plasmid preparation (DNA construct) and transfection

To establish the (*AID-EWSR1/AID-EWSR1*) DLD-1 cell line, CRISPR/Cas9 system was employed to tag the 5’ end of *EWSR1* gene with *mini-AID (mAID)*.

The donor plasmid of *mAID* was generated by following the procedure of the previous report with a minor modification (Hassebroek et al., 2020). First, the homology arms (left/up and right/down) that targets the *EWSR1* locus were amplified from genomic DNA extracted from DLD-1 using following primers, respectively. Note that mutations were introduced in PAM sequences on the homology arms to avoid the Cas9 dependent degradation of the donor plasmid. The PCR products for the homology arms were inserted into the donor plasmid (containing hygromycin resistant gene/P2A sequence and 3x mini-AID and 3X Flag sequence) using *PciI/SalI* and *SpeI/NotI* sites, respectively (Hassebroek et al., 2020).

The guide RNA sequence was designed using the CRISPR Design Tool; Zhang laboratory, MIT. The suggested guide RNA sequences are listed below.

- *EWSR1* gRNA1 F: ATG​GCG​TCC​ACG​GGT​GAG​TA
- *EWSR1* gRNA1 R: TAC​TCA​CCC​GTG​GAC​GCC​AT
- *EWSR1* gRNA2 F: AGT​TCC​ACC​ATA​CTC​ACC​CG
- *EWSR1* gRNA2 R: CGG​GTG​AGT​ATG​GTG​GAA​CT

The oligonucleotides (synthesized by Integrated DNA technologies, IDT) were annealed, inserted into pX330 at its BbsI site (Addgene, #42230), and its sequence was confirmed by the DNA sequencing (ACGT Inc.).

The *EWSR1-mCherry* and *EWSR1:R565A-mCherry* DNA constructs that targets the *AAVS1* locus of the (*AID-EWSR1/AID-EWSR1*) DLD-1 cell line are generated as described below. The *mCherry*, *hEWSR1,hEWSR1:R565A* genes were individually amplified using *pCDNA4-His-maxC-mCherry*, *pSG5-2XFLAG-hEWSR1* or *pSG5-2XFLAG-hEWSR1:R565A* plasmids as a template using the following primers (Park et al., 2014).

- *MluI-hEWS* F: 5′-GAT​ACG​CGT​ATG​GCT​GCC​ACG​GAT​TAC-3′
- *NotI*nostop3′h*uEWS* 1965to1945*hEWS* R: 5′-CTG​CGG​CCG​CGT​AGG​GCC​GAT​CTC​TGC-3′
- *NotI mChe* F: 5′-GCG​GCC​GCA​GGC​GCT​GG-3′
- *SalI stop mChe* R: 5′-CTT​GTC​GAC​TTA​CTT​GTA​CAG​CTC​GTC​C-3′

The PCR products of *hEWSR1*, *hEWSR1:R565A* and *mCherry* were inserted to *SalI* and *MluI* sites of pMK243 that had been modified at its multicloning sites in the previous study (Tet-OSTIR1-PURO) (Natsume et al., 2016; Hassebroek et al., 2020; Park et al., 2021).

### Establishment and maintenance of the stable cell lines

To establish the (*AID-EWSR1/AID-EWSR1*) DLD-1 cell line, the DLD1-OsTIR cells plated in a 3.5 cm dish were transfected with the *mAID*-donor and guide RNA (gRNA) plasmid constructs using ViaFect (Promega, #E4981) (Russell et al., 2010; Mali et al., 2013; Hassebroek et al., 2020). The transfected cells were cultured for two days, and split to 10 cm dishes. Next, the cells were further cultured in the medium containing 1 μg/mL Blasticidin (Invivogen, #ant-bl) and 200 μg/mL Hygromycin B Gold (Invivogen, #ant-hg) for 10–14 days until they formed colonies. Sixteen colonies were isolated, and the integration of transgenes were verified with PCR using the genomic DNA obtained from each clone using the following primers.

- *hEWSR1* GenPCR 5′ F: 5′-CCC​GGG​TAC​TCA​CTG​CAC​GAG-3′
- *hEWSR1* GenPCR 3′ R: 5′-CGG​CTT​GGG​GCT​GGA​AGC-3′

Among sixteen clones, we chose clone #19, a homozygous (*AID-EWSR1/AID-EWS*R1) clone, for subsequent analysis (referred to as OsTIR7-19 in the following section). Conditional degradation of mAID tagged EWSR1 protein in the AUX treated OsTIR7-19 cells was confirmed by immunocytochemistry and western blotting analysis using anti-FLAG (Sigma, #F7425) and anti-EWSR1 (Santa Cruz, #sc-48404), respectively.

To establish the (*AID-EWSR1/AID-EWSR1;EWSR1-mCherry*) and (*AID-EWSR1/AID-EWSR1;EWSR1:R565A-mCherry*) DLD-1 cell lines, Tet-On transgene that contains *EWSR1-mCherry* or *EWSR1:R565A-mCherry* were integrated into the safe harbor *AAVS1* locus of the OsTIR7-19 (*AID-EWSR1/AID-EWSR1*) DLD-1 cell line. The OsTIR7-19 cells were plated in a 3.5 dish, and transfected with the donor and guide RNA plasmids targeting *AAVS1* locus (Addgene, AAVS1 T2 CRISPR in pX330, #72833) (described in the previous section) using ViaFect™ (Promega, #E4981). The cells were cultured for 2 days, re-plated in 10 cm dishes, and were selected for ten to 14 days with a selection medium (contains 1 μg/mL Blasticidin [Invivogen, #ant-bl], 200 μg/mL Hygromycin B gold [Invivogen, #ant-hg] and 1 μg/mL Puromycin). Twenty four clones were screened for the mCherry signal visually by fluorescence microscopy, and the integration of transgene in its genome was confirmed by the genomic PCR using the following primers.

- *AAVS1* F: 5′-CTG​CCG​TCT​CTC​TCC​TGA​GT-3′
- Pause Site R: 5′-GTT​TTG​ATG​GAG​AGC​GTA​TGT​TAG​TAC-3′
- *SV40* F: 5′-CCG​AGA​TCT​CTC​TAG​AGG​ATC​TTT​GTG​AAG-3′
- *AAVS1* R: 5′-CAA​AAG​GCA​GCC​TGG​TAG​AC-3′

The concentration of antibiotics used in this study are 1 ug/mL Puromycin (PUR^R^), Invivogen), 1 μg/mL Blasticidin (BSD^R^) (Invivogen, #ant-bl) and 200 μg/mL Hygromycin B (HYG^R^) gold (Invivogen, #ant-hg). The (*AID-EWSR1/AID-EWSR1*) cells were maintained in the McCoy’s 5A medium that contains BSD and HYG. The (*AID-EWSR1/AID-EWSR1;EWSR1-mCherry*) and (*AID-EWSR1/AID-EWSR1;EWSR1:R565A-mCherry*) DLD-1 cells were maintained in the McCoy’s 5A medium with BSD, HYG and PUR.

To knockdown the AID-EWSR1, the cells were treated with 500 µM indole-3-acetic acid (3-IAA, or AUX) for 24 h. To express exogenous EWSR1-mCherry or EWSR1:R565A-mCherry, both cell lines were treated with 1 μg/mL of doxycycline (DOX) for 24 h.

The expression of EWSR1-mCherry and EWSR1:R565A-mCherry proteins were confirmed by immunocytochemistry using rat anti-RFP (1:1,000 dilution) (Bulldog Bio Inc., #RMA5F8) followed by anti-Rat Alexa Fluor 568 (1:500 dilution) (Invitrogen, #A-11077), and by western blotting using chicken anti-mCherry antibody (1:1,000 dilution, LSBio, #C-204825), followed by IRDye 800 CW donkey anti-chicken IgG secondary antibody (1:10,000 dilution) (LI-COR, #926-32218).

### Western blotting

Whole cell lysates obtained from cells with/without AUX/DOX were subjected to western blotting. Primary antibodies used in this study are; mouse anti-C9 EWS antibody (1:1,000 dilution) (Santa Cruz, #sc-48404); rabbit anti-FLAG antibody (1:1,000 dilution) (Sigma, #F7425); rabbit anti-CyclinB antibody (1:1,000 dilution, Sigma, #C8831); mouse anti-β-actin antibody (1:1,000 dilution, Sigma, #A2228); chicken anti-mCherry antibody (1:1,000 dilution, LSBio, #C-204825). Secondary antibodies used in this study are; IRDye 680RD donkey anti-mouse IgG (1:10,000 dilution, LI-COR, #926–68072), IRDye 800CW donkey anti-rabbit IgG (1:10,000 dilution, LI-COR, #926-32213), IRDye 800CW donkey anti-mouse IgG (1:10,000 dilution, LI-COR, # 926-32212), IRDye 800CW donkey anti-chicken IgG (1:10,000 dilution, LI-COR, # 926-32218). All images of western blotting were captured by LI-COR Odyssey Imaging System.

### Chromosome spread

The cells grown in a T25 flasks at ∼70%–80% confluency were synchronized to mitosis using the thymidine/nocodazole protocol (Kotýnková et al., 2016; Park et al., 2021). The mitotic cells were collected in a 15 mL tube, washed for 3 times with McCoy’s 5A media (without FBS), and it were resuspended in 1 mL of PBS. Half (500 µL) of the mitotic cell suspension was transferred to new tube by mixing with 1 mL of dH_2_O, and it was incubated for 5 min at room temperature. Then, the mitotic cell suspension solution (250 µL) was loaded into a cytology funnel (BMP, #CYTODB25), and it was subjected to cytospin for 5 min at 1,000 rpm with max acceleration (Thermo Shandon Cytospin3 Centrifuge, #TH-CYTO3). The slides were subjected to immunocytochemistry as described in the following section.

### Immunocytochemistry

The mitotic cells were subjected to immunocytochemistry by following the protocol described in the previous report (Park et al., 2014). The antibodies used in this study are; Mouse anti-C9EWS antibody (1:500 dilution) (Santa Cruz, #sc-48404); rabbit anti-FLAG antibody (1:500 dilution) (Sigma; rabbit anti-Aurora B antibody (1:500 dilution) (abcam,#ab2254); guinea pig anti-CENP-C antibody (1:500 dilution) (MBL, #PD030); mouse anti-α-tubulin antibody (1:4,000 dilution) (Sigma, #T8328); anti-mouse Alexa fluor 568 (1:500 dilution) (Invitrogen,# A-11004); Rat anti-RFP (1:1,000 dilution) (Bulldog Bio Inc., #RMA5F8), anti-rabbit Alexa fluor 488 (1:500 dilution) (Invitrogen, #A32790); anti-guineapig Alexa fluor 568 (1:500 dilution) (Invitrogen, #A-21450) anti-rat Alexa Fluor 568 (1:500 dilution) (Invitrogen, #A-11077).

The chromosomes spread on a slide was fixed with 100 uL of 4% Paraformaldehyde (PFA) at room temperature for 5 min, washed for three times with 0.01% Triton X-100/Phosphate Buffered Saline (PBS) for 5 min at room temperature, respectively, permeabilized with ice-cold methanol for 5 min at −20°C, and washed for three times with PBS for 5 min at room temperature. Then, the chromosomes on the slide was treated with 100 ul of blocking solution (1% Fetal Bovine Serum (FBS)/PBS) at room temperature for 30 min. The slides were subjected to immunocytochemistry with following primary antibodies; rabbit-anti-Aurora B antibody (1:500 dilution) (Abcam, #ab2254) guineapig-anti CENP-C antibody (1:500 dilution) (MBL, #PD030), rat-anti RFP (1:500 dilution) (Bulldog Bio Inc., #RMA5F8) for over-night at 4°C. Next day, the chromosomes spread on the slide were washed three times with PBS at room temperature for 5 min by repeating three times, and incubated with following secondary antibodies; anti-Rabbit Alexa Fluor 488 (1:500 dilution) (Invitrogen, # A32790), anti-guineapig Alexa Fluor 647 (1:500 dilution) (Invitrogen # A-21450), or anti-rat Alexa Fluor 568 (1:500 dilution) (Invitrogen, #A-11077) for 1 h at room temperature, washed three times with PBS at room temperature for 5 min by repeating three times, and mounted in VECTASHILED Antifade Mounting Medium with DAPI (Vector laboratory, #H-1200).

### Aneuploidy analysis

The cells were plated at ∼30% confluency, followed by the treatment with/without AUX/DOX for 48 h. Concurrently, the cells were treated with thymidine/nocodazole, and the mitotic cells were subjected to the cytospin procedure as described in the previous section. Mitotic chromosomes were visualized with following antibodies; rabbit-Topo2A antibody (gift from Dr. Yoshiaki Azuma) followed by Alexa flour 488 anti-rabbit secondary antibody; rat-RFP antibody followed by Alexa flour 568 anti-rat secondary antibody (Invitrogen); anti-guineapig CENP-C antibody (MBL) followed by Alexa Flour 647 anti-guineapig antibody (Invitrogen). All antibodies were diluted at 1:500 dilution rate. The images were adjusted using the linear function curve of Photoshop.

### Image documentation

The images of cells and chromosomes were acquired using the Nikon Plan Apo 60x or 100×/1.4 oil objective lens on a TE2000-U microscope (Nikon) with a Retiga SRV CCD camera (QImaging) operated with Meta Morph imaging software (MetaMorph Inc.) at room temperature. In addition, the confocal images of cells were documented with Leica TCS SPE Laser Scanning Confocal DM6-Q microscope using the ACS Apo 63x/Oil objective lens operated by Leica LAS X Imaging software.

### Statistical analysis

All graphs are presented as mean with standard deviation (S.D.) or standard error of the mean (S.E.M). The statistical analysis was conducted with one-way or two-way ANOVA followed by Tukey’s multiple comparison test or two-tailed paired t-test using GraphPad9 software (confidence was defined at *p* < 0.05).

## Results

### The EWSR1 regulates faithful chromosomal segregation

**FIGURE 1:**
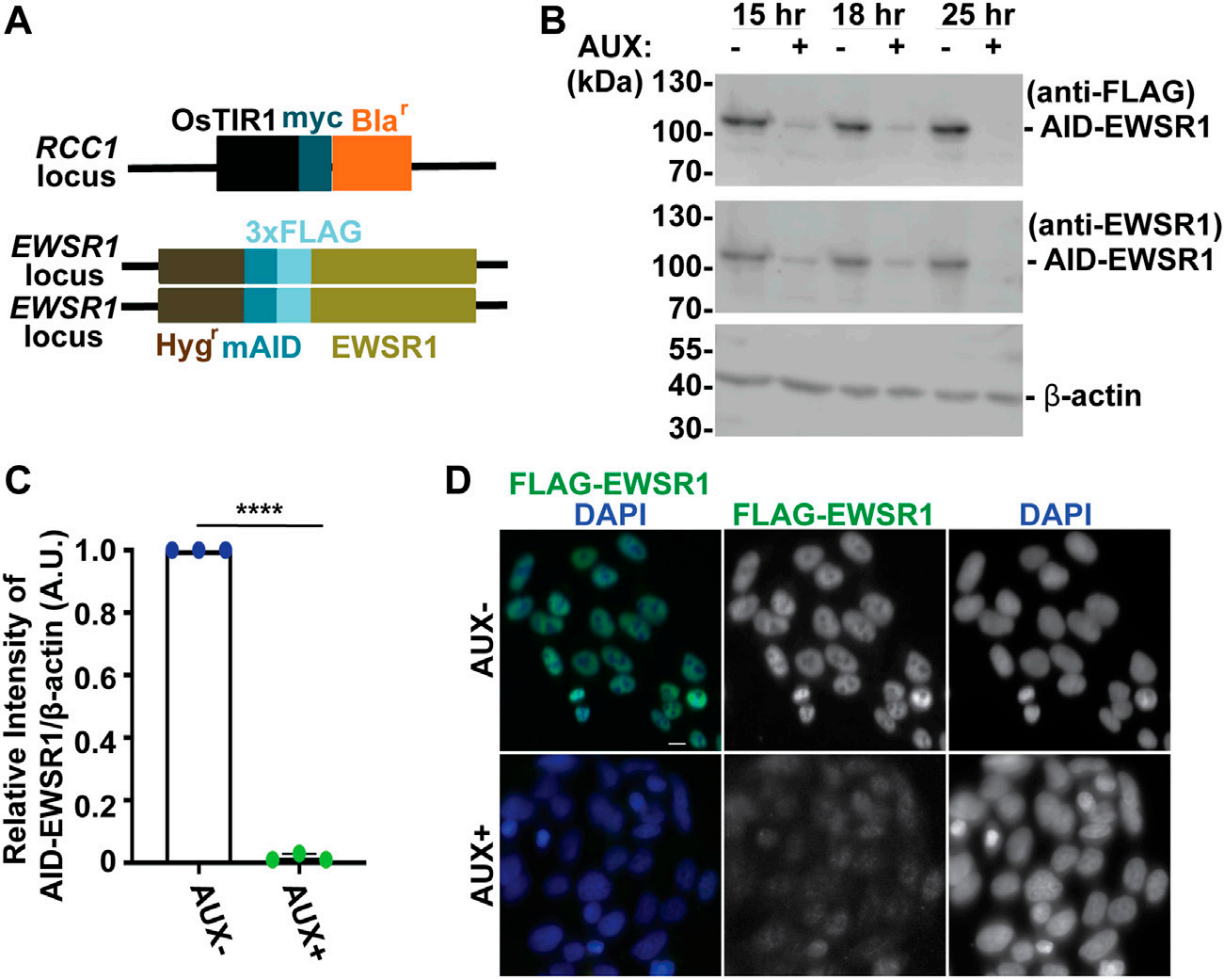
The treatment of a stable *AID-EWSR1/AID-EWSR1* DLD-1 cell line with Auxin induces an efficient degradation of AID-EWS. **(A)**. Schematic diagram of *OsTIR1* and *AID-EWSR1* DNA construct. *OsTIR1*: plant driven E3 ligase, *Bla* ^*r*^: Blasticidine resistance gene, *Hyg* ^*r*^: Hygromycin resistance gene, *mAID*: mini Auxin-Inducible Degron tag, *3XFLAG*; three tandem repeat of FLAG tag. The *OsTIR1* construct was inserted at the *RCC1* locus, and *mAID* construct was inserted to both *EWSR1* loci (homozygous). **(B)**. Representative images of western blotting using anti-FLAG (top panel), anti-EWSR1 (middle panel) and anti-β actin (bottom panel) obtained from AUX- and AUX + cells in the presence of Auxin at given time point (15 h, 18 h and 25 h). **(C)**. Relative intensity of the bands of western blotting visualized with anti-EWSR1 (normalized to the bands with anti-β-actin) obtained from AUX- and AUX + cells (*n* = 3 experiments). Graph shows the mean of each group with Standard Deviation (SD). (*n* = 3 experiments). ^****^ *p* < 0.0001 (Two tailed paired *t*-test). **(D)**. Representative images of immunocytochemistry using anti-FLAG (green) and DAPI (blue) obtained from AUX- and AUX + cells. Scale bar = 10 um.

To determine whether loss of EWSR1 induces aneuploidy after 1 cell cycle, or cells with aneuploid expand during a long period of time, it is essential to control the timing of the knockdown of EWSR1. To accomplish this, we employed the Auxin-Inducible Degron (AID) system to degrade EWSR1 conditionally in DLD-1 cells (Yesbolatova et al., 2019). The major reason for using this conditional knockdown system is that it allows us to assess the effect of EWSR1 within a single cell cycle, thus the phenotypic change caused by the knockdown of protein of interest leads to a better understanding of the activity of the protein. The CRISPR/Cas9 DNA constructs, a *mAID-3xFLAG-Hyg* ^*r*^ (Hyg, Hygromycin resistant gene) and two guide RNA constructs that target the start codon of the *EWSR1* gene, were transfected into the stable DLD-1 cell line that expresses a plant derived E3 ligase*, OsTIR1* (integrated at a *RCC1* locus) (Figure 1A) (Hassebroek et al., 2020). The DLD-1 cell line was used in this study because it has a near-diploid karyotype, and it has been used to model the activity of proteins of interest in aneuploidy induction (Lengauer et al., 1997). The cells transfected with the CRISPR/Cas9 DNA constructs were selected with hygromycin, and the genomic DNAs of the colonies were amplified by PCR to verify the integration of the *mAID-3xFLAG-Hyg* ^*r*^ construct. Among a total of 48 colonies that were screened, 11 clones carried the *AID-EWSR1/wt* genotype, and 5 clones carried the *AID-EWSR1/AID-EWSR1* genotype (Supplementary Figure S1)*.* Among the *AID-EWSR1/AID-EWSR1* homozygous clones, we chose clone #19 to assess the function of EWSR1 in the following study.

We began our study by characterizing the Auxin dependent degradation of EWSR1 in the newly established (*AID-EWSR1/AID-EWSR1*) DLD-1 cell line. Because the cell cycle time of DLD-1 cells is approximately 22 h, the cells were treated with 500 µM of Auxin (AUX+) for 15, 18 and 25 h, and they were subjected to western blotting using anti-FLAG and anti-EWSR1 antibodies to assess the level of EWSR1 degradation (Dexter et al., 1979). EWSR1 protein was maximally degraded in cells treated with AUX for 25 h accomplished maximum degradation of EWSR1 (Figure 1B). Repetition of the experiment three times revealed a consistent reduction of the signal of EWSR1 protein (normalized by β-actin) in the AUX + sample when it was compared to the AUX-sample (****p < 0.0001) (Figure 1C). Based on this result, the cells were treated with AUX for a minimum of 24 h in the following experiments. In addition, immunocytochemistry using anti-FLAG antibody confirmed drastic reduction of the FLAG-EWSR1 protein in the AUX treated (AUX+) cells (Figure 1D). Together, treatment of *AID-EWSR1/AID-EWSR1* cells with AUX leads to efficient degradation of EWSR1.

**FIGURE 2:**
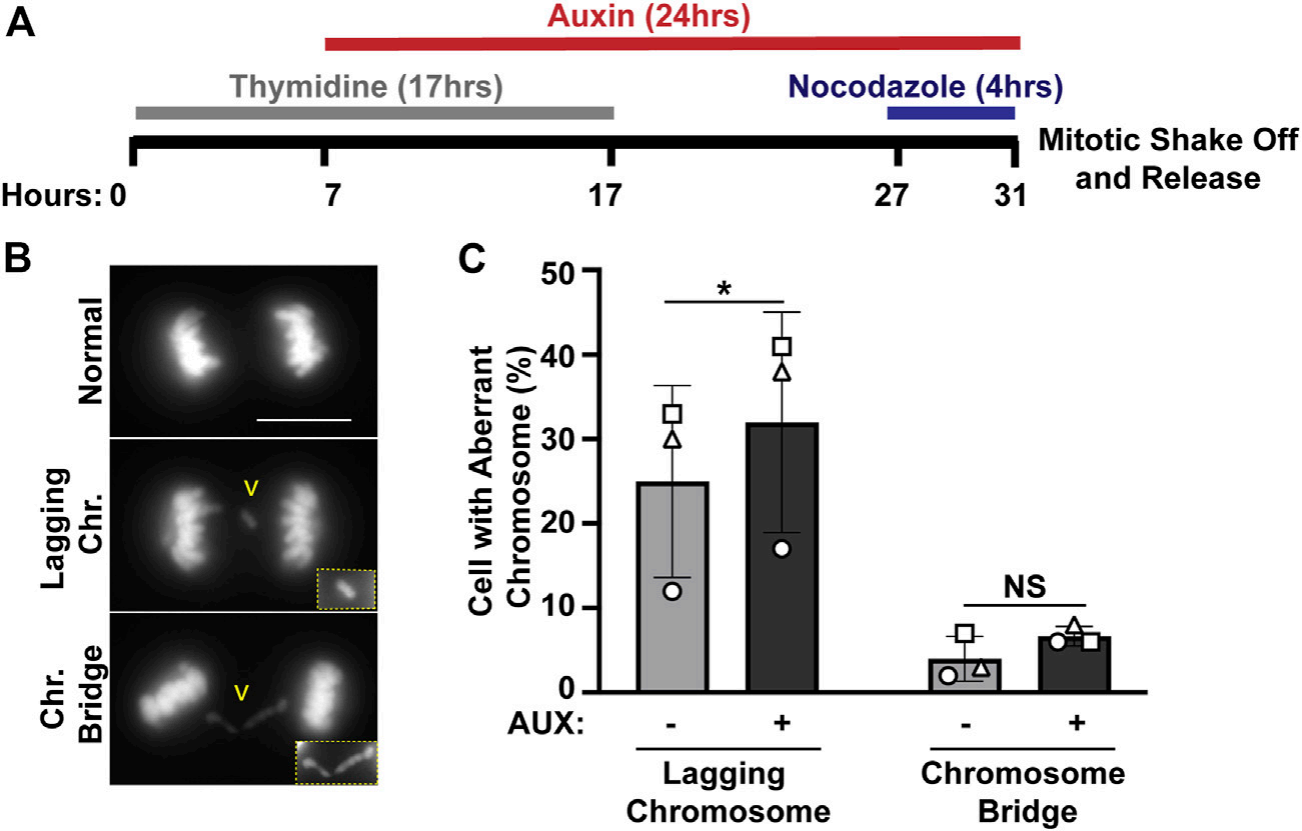
The EWSR1 knockdown promotes induction of lagging chromosome during anaphase. **(A)**. Schematic diagram for the mitotic synchronization of EWSR1 knockdown cells using Thymidine and Nocodazole, followed by the mitotic shake-off and release from the mitotic arrest for 60 min. **(B)**. Representative images of chromosomes; normal (top panel), lagging chromosome (middle panel) and chromosome bridge (bottom panel). Yellow square highlights the magnified images of either lagging chromosome or chromosome bridge. Chr.; chromosome, Scale bar = 10 um. **(C)**. Percentages of anaphase cells with lagging chromosomes (left graph) and of chromosomal bridges (right graph) in AUX- and AUX + cells (Total 34-104 anaphase cells per sample, *n* = 3 experiments). *p < 0.05. (paired t-test), NS; Non-Significant.

We previously identified *ewsr1a and ewsr1b* in zebrafish (the homologues of human *EWSR1*), and proposed that the proteins regulate faithful chromosomal segregation during anaphase (Azuma et al., 2007). The analysis was done in 24 h post-fertilization (hpf) zebrafish (an embryonic stage when basic structures of organs including brain, and somites are established), thus the study did not address whether the defects were directly derived from a single cell cycle, or from secondary defects derived from multiple cell cycles. To elucidate this question, the stable EWSR1 knockdown cell line was treated with AUX, and the incidence of aberrant chromosome segregation in the EWSR1 knockdown (AUX+) cells was compared to the control (AUX-) cells. Both AUX- and AUX + cells were first synchronized in mitosis using the conventional thymidine/nocodazole protocol, and the samples were fixed at 60 min after the release from nocodazole (Figure 2A). It is noteworthy that the treatment of both AUX- and AUX + cells with nocodazole (a chemical that interferes with polymerization of tubulin) successfully synchronized both cells at prometaphase, suggesting that the Spindle Assembly Checkpoint (SAC) in both sample groups is active. Therefore, it is likely that EWSR1 does not regulate SAC. The chromosomes of AUX- and AUX + cells were visualized with DAPI, and the incidences of aberrant segregation of chromosomes, lagging chromosomes and chromosome bridges, were scored. The AUX + cells displayed significantly higher incidence of lagging chromosomes compared to the AUX-cells (Figures 2B, C). In constrast, although the incidence of chromosome bridges in AUX+ cells was slightly higher than in AUX− cells the difference was not statistically significant (*n* = 3 experiments) (Figures 2B, C). Note that the incidence of chromosome bridges in AUX + cells was slightly increased compared to AUX-cells. Together, the results suggests that EWSR1 maintains chromosomal stability by reducing the frequency of lagging chromosomes.

One possible explanation for the induction of lagging chromosomes in EWSR1 knockdown (AUX+) cells is that the defect is caused by misalignment of chromosomes during metaphase. Therefore, the percentages of misalignment metaphase chromosomes (assymmetric and/or “left-over” chromosomes visualized with DAPI signals) were compared between two sample groups (AUX- and AUX + cells), and the result showed that there was no significant difference between the two groups (Supplementary Figures S2A, B).

### The EWSR1 protein is required for the proper localization of Aurora B at inner centromeres

In mitosis microtubules nucleated by the two centrosomes, one at each pole, attach to kinetochores. There are two kinetochores at each centromere, one on each chromatid. To accomplish faithful chromosome segregation during mitosis, microtubules nucleated from centrosome have to attach to the kinetochore on one chromatid and microtubules from the other centrosome have to attach to the kinetochore on the other chromatid. If microtubules from only one centrosome attach to both kinetochores (syntelic attachment) or if most microtubules are correctly attached but some microtubules from both centrosomes connect to the same kinetochore (merotelic attachment), the result will be lagging chromosomes during anaphase (Cimini et al., 2001a). Prior to anaphase, Aurora B kinase, the enzymatic subunit of the chromosome passenger complex (CPC) is recruited to the site of attachment errors where it destabilizes kinetochore-microtubule interactions facilitating error correction (Ohi et al., 2003). Thus, impairment of Aurora B localization or function is a strong candidate mechanism by which knockdown of EWSR1 might cause lagging chromosomes. To study how Aurora B is affected in the EWSR1 knockdown cells, we first addressed whether there is a change in the expression levels of Aurora B between AUX- and AUX + cells by western blotting using anti-FLAG antibody (to visualize EWSR1), anti-Aurora B, and anti-β-actin (loading control) (Supplementary Figure S3A). Intensity of the AID-EWSR1 and the Aurora B bands were measured (*n* = 3 experiments), and were normalized by the intensity of β-actin (Supplementary Figures S3B, C). Consistent from the result shown in Figure 1, the cells treated with auxin (AUX+) cells displayed degradation of EWSR1 proteins, whereas there was no significant difference in the intensity of Aurora B signals between the two sample groups (Supplementary Figures S3B, C). These results suggest that the EWSR1 does not regulate the expression of Aurora B protein. Furthermore, the same experiment using anti CENP-C antibody was conducted, and there was no significant differences of the protein levels of CENP-C between AUX- and AUX + cells (Supplementary Figures S3D–F). This suggests that EWSR1 does not regulate the expression of CENP-C protein.

**FIGURE 3:**
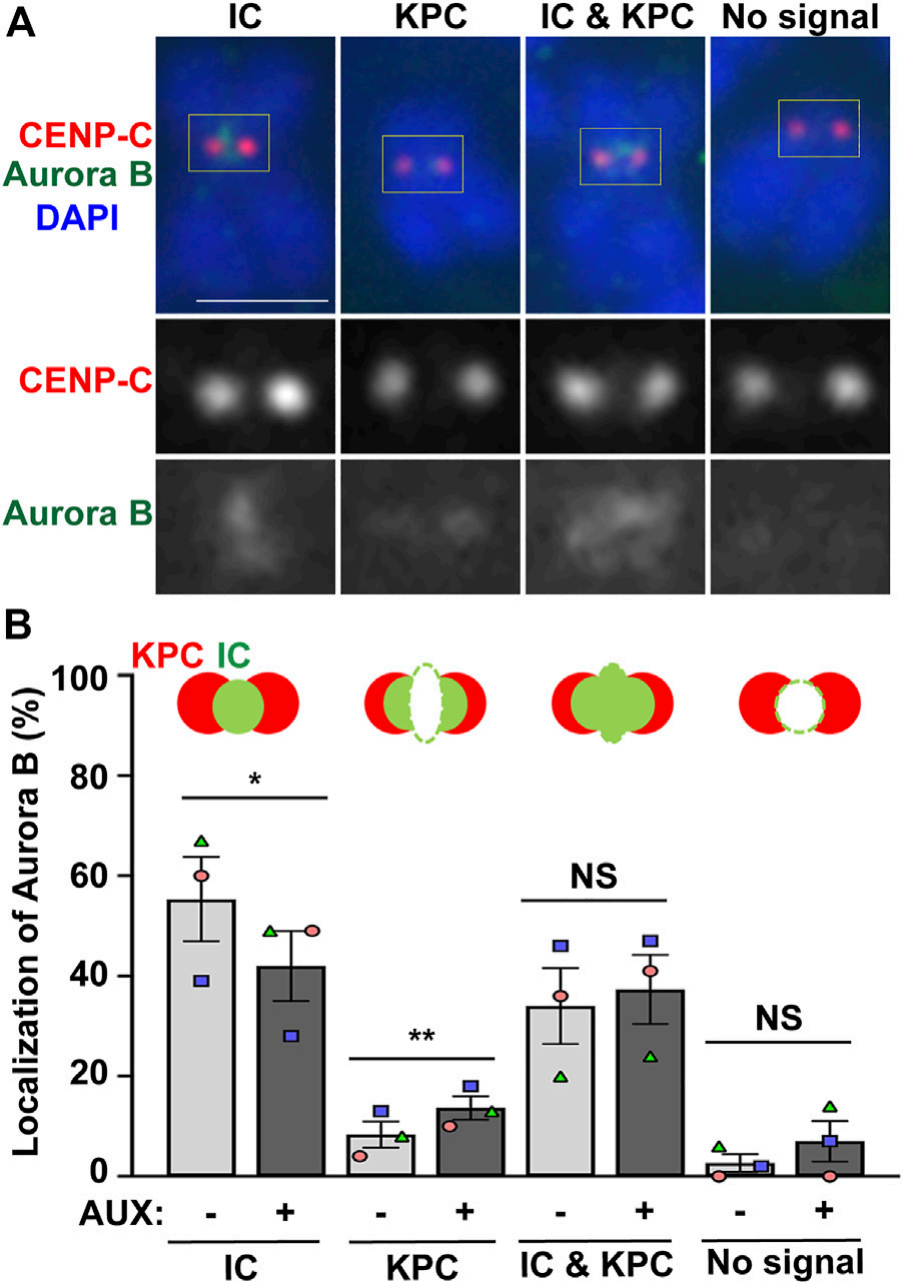
EWSR1 contributes to for Aurora B localization at inner centromere, and prevents its localization at kinetochore during early mitosis. **(A)**. Representative images of Aurora B on pro/metaphase chromosome obtained from control (AUX-) cells that were released from thymidine/nocodazole for 30 min. The Aurora B (Green, top and bottom panel) and CENP-C (kinetochore marker) (Red, top and middle panel) were visualized by immunocytochemistry using anti-Aurora B and anti-CENP-C antibodies. Classification of the localization of Aurora B; Inner Centromere (IC), Kinetochore Proximal Centromere (KPC), Inner Centromere and Kinetochore Proximal Centromere (IC + KPC), and No Signal. Scale bar = 10 um. **(B)**. Quantification of Aurora B localization at IC, KPC, IC + KPC, and No signal between AUX- and AUX + cells (total of 712–732 chromosomes per sample, *n* = 3 experiments). Values are mean with standard error of the mean (SEM). Two-tailed paired t-test; *p < 0.05, **p < 0.01, NS; Non-Significant.

Next, we examined whether the localization of Aurora B is altered in the EWSR1 knockdown (AUX+) cells. The cells were synchronized at prometaphase using thymidine/nocodazole treatment, released for 30 min, followed by the cytospin procedure to spread the cells on a slide. Then, mitotic chromosomes on the slides were subjected to immunocytochemistry using anti-Aurora B (green) and a centromeric marker, anti-CENP-C (red). The localization of Aurora B was scored and the localization patterns were divided into four groups, localization at the inner centromere only, kinetochore proximal centromere (KPC) only, both inner centromere and KPC and no signal (Figure 3A). There was a significantly decreased incidence of Aurora B at inner centromeres, and an increased incidence of the localization of Aurora B at KPC in the EWSR1 knockdown cells (AUX+) compared to those of control cells (AUX-) (Figure 3B). The data suggests that EWSR1 plays a significant role in the localization of Aurora B at the inner centromere.

To further address whether the activity of Aurora B is impaired in the EWSR1 knowckdown cells, the phosphorylation status of the Histone H3 at Ser 28 (a known phosphorylation site of Aurora B) was compared between the AUX- and AUX + cells by western blotting. There was no significant difference of the intensity of Histone H3 between the two sample groups, whereas the levels of the phosphorylated Histone H3 at Ser 28 was significantly reduced in the AUX + cells compared to AUX-cells (*n* = 3 experiments) (Supplementary Figures S4A, B). These results suggest that the EWSR1 is required for the canonical Aurora B activity.

One possible mechanism for the EWSR1-dependent regulation of Aurora B is that the EWSR1 localizes to the centromere/kinetochore-proximal region, and prevents the induction of lagging chromosomes. Our previous localization study for EWSR1 in A673 and HeLa cells did not identify a strong signal/foci on the chromosomes, presumably due to the limited resolution of the microscope or due to the cell type specific phenomenon (Park et al., 2014). The localization of EWSR1 in DLD-1 cells during mitosis had not been examined before, thus, we aimed to study whether the EWSR1 localizes at centromere/kinetochore-proximal region during prometa/metaphase. To avoid the possibility of obtaining antibody-derived background signals, we generated a DLD-1 cell line that has an *mNEON* gene tag at the 5′end of *EWSR1* alleles (Supplementary Figure S5A). The DLD-1 cells transfected with the mNEON CRISPR/Cas9 DNA construct targeting the *EWSR1* locus underwent selection process with zeomycine containing medium. Among the selected clones, clone #12 was identified as a homozygous (*mNEON*-*EWSR1/mNEON*-*EWSR1*) DLD-1 line. Its endogenous mNEON-EWSR1 signals resembles the localization patterns of EWSR1 visualized with EWSR1 antibody (Supplementary Figures S3B–D) (Park et al., 2014)*.* Based on these data, clone #12 was used in the following study.

**FIGURE 4:**
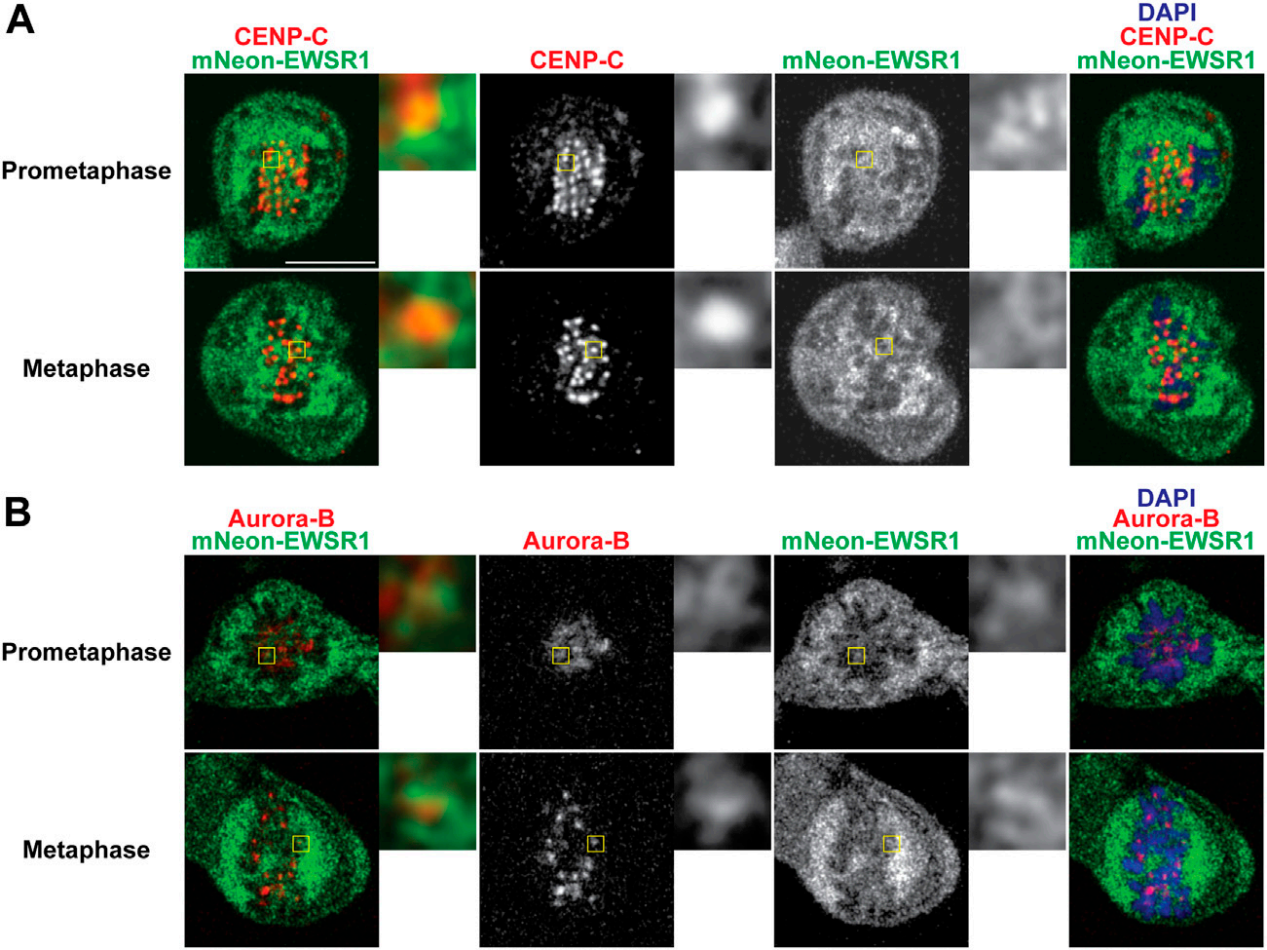
EWSR1 partially colocalizes with CENP-C and Aurora B at the kinetochore proximal region in DLD-1 cells. **(A)** Merged images with CENP-C (Red), mNEON-EWSR1 (Green), and DNA stained using DAPI (blue). CENP-C (red) was visualized using an anti-CENP-C antibody, and mNEON-EWSR1 is shown in green (Left panel); CENP-C (Red) and mNEON-EWSR1 (Green), CENP-C (Red), mNEON-EWSR1 (Green), and DAPI (Right panel). **(B)** Single Z-section images with Aurora B (Red), mNEON-EWSR1 (Green), and DNA stained using DAPI (blue). Aurora B (red) visualized using anti- CENP-C antibody, and mNEON-EWSR1 (green) (Left panel); Aurora B (Red) and mNEON-EWSR1 (Green), Aurora B (Red), mNEON-EWSR1 (Green), and DAPI (Right panel). Scale bar 10 um.

To elucidate whether mNEON-EWSR1 colocalizes with CENP-C, the (*mNEON*-*EWSR1/mNEON*-*EWSR1*) DLD-1 line was subjected to immunocytochemistry using anti-CENP-C, and the cells were photo-documented with a confocal microscope. Detailed observations using sets of Z-stack images revealed that all CENP-C signals (red) overlaps with a part of mNEON-EWSR1 signal (green) in prometaphase (*n* = 10 cells) and metaphase (*n* = 10 cells) (Figure 4A). Therefore, the data indicates that EWSR1 localizes at kinetochore-proximal region. To study whether EWSR1 and Aurora B colocalizes, the (*mNEON*-*EWSR1/mNEON*-*EWSR1*) DLD-1 line was further subjected to immunocytochemistry using anti-Aurora B. The analysis with all Z-section images and 3D constructed images of a single cell images reveled that all Aurora B foci located on chromosomes overlapped with a part of mNEON-EWSR1 signal during prometaphase (*n* = 10 cells) and metaphase (*n* = 10 cells) (Figure 4B). Together, EWSR1 co-localizes with Aurora B at centromere/kinetochore region during prometaphase and metaphase.

### The EWSR1 knockdown cell does not undergo mitotic arrest

**FIGURE 5:**
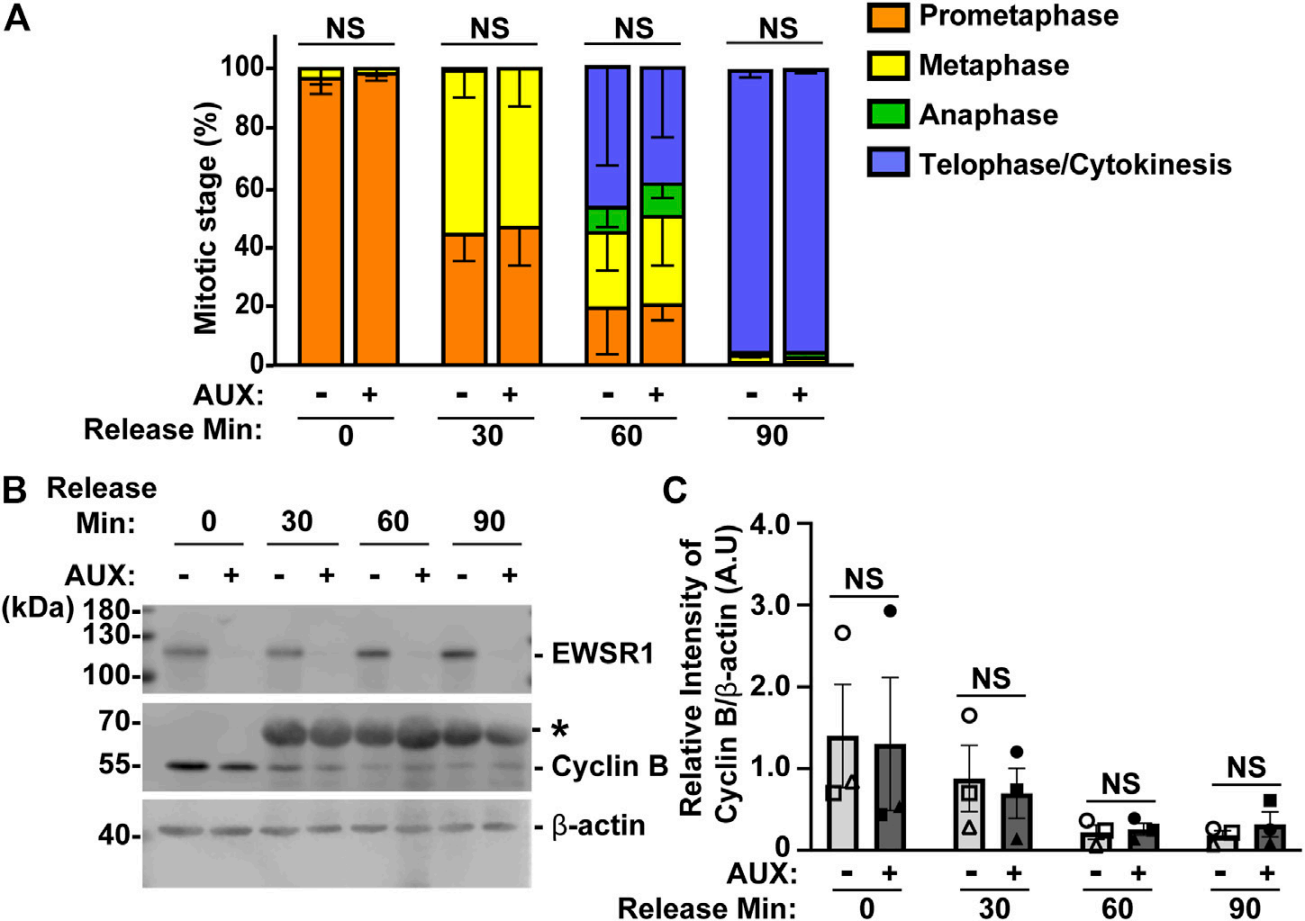
The EWSR1 knockdown cells do not arrest at any mitotic stages. **(A)**. The percentages of mitotic stage of AUX- and AUX + cells released for 0, 30, 60 and 90 min after the thymidine/nocodazole treatment (total of 83–173 cells per sample, *n* = 3 experiments). NS = non-significant. **(B)**. Representative images of western blotting using anti-FLAG (top panel), anti-Cyclin B (middle panel) and anti-β actin (bottom panel) obtained from AUX- and AUX + cells released at 0, 30, 60, and 90 min after the thymidine/nocodazole treatment. *: Non-specific band. **(C)**. Quantification of the levels of Cyclin B protein (normalized by β-actin) obtained from AUX- and AUX + cells (*n* = 3 experiments). Images of western blotting gel using anti-EWSR1 (top panel), anti-Cyclin B (middle panel), and anti-β-actin antibodies (bottom panel). Values are mean with S.D. using two-way ANOVA with Tukey’s multiple comparison test. NS: Non-Significant.

In general, when the process of chromosomal segregation is impaired, the cell should be arrested at mitosis so that the error can be corrected. Cells are arrested before anaphase onset when microtubules are incorrectly connected to kinetochores, or before cytokinesis when chromosomes lag or bridges occur during anaphase. Because the EWSR1 knockdown (AUX+) cells displayed high incidence of lagging chromosomes, we aimed to determine whether the EWSR1 knockdown (AUX+) cells underwent mitotic arrest. The cells were treated with or without Auxin (AUX- and AUX+), and the cells were synchronized in mitosis using the same thymidine/nocodazole protocol described in Figure 2A. The cells arrested and plated on a coverslip at prometaphase (0 min), released for 30 min, 60 min and 90 min. The stages of mitosis were scored based on the shape of chromosomes (DAPI signal), and its incidences were compared between AUX- and AUX + cells. To our surprise, there were no significant differences between the duration of the stages of mitosis of AUX- and AUX + cells nor the length of mitosis (Figure 5A).

To further verify the result, we employed a biochemical approach to quantify the level of Cyclin B, a protein that is known to undergo degradation during anaphase (Minshull et al., 1990). The results showed that there was no significant difference in the level of Cyclin B proteins between AUX- and AUX + cells in all time course samples (Figures 5B, C). Despite the fact that the EWSR1 is required for faithful chromosomal segregation, the results suggest that EWSR1 knockdown does not affect the duration of mitosis by activating checkpoint controls.

### The EWSR1 protein prevents the induction of aneuploidy through interaction with Aurora B

**FIGURE 6:**
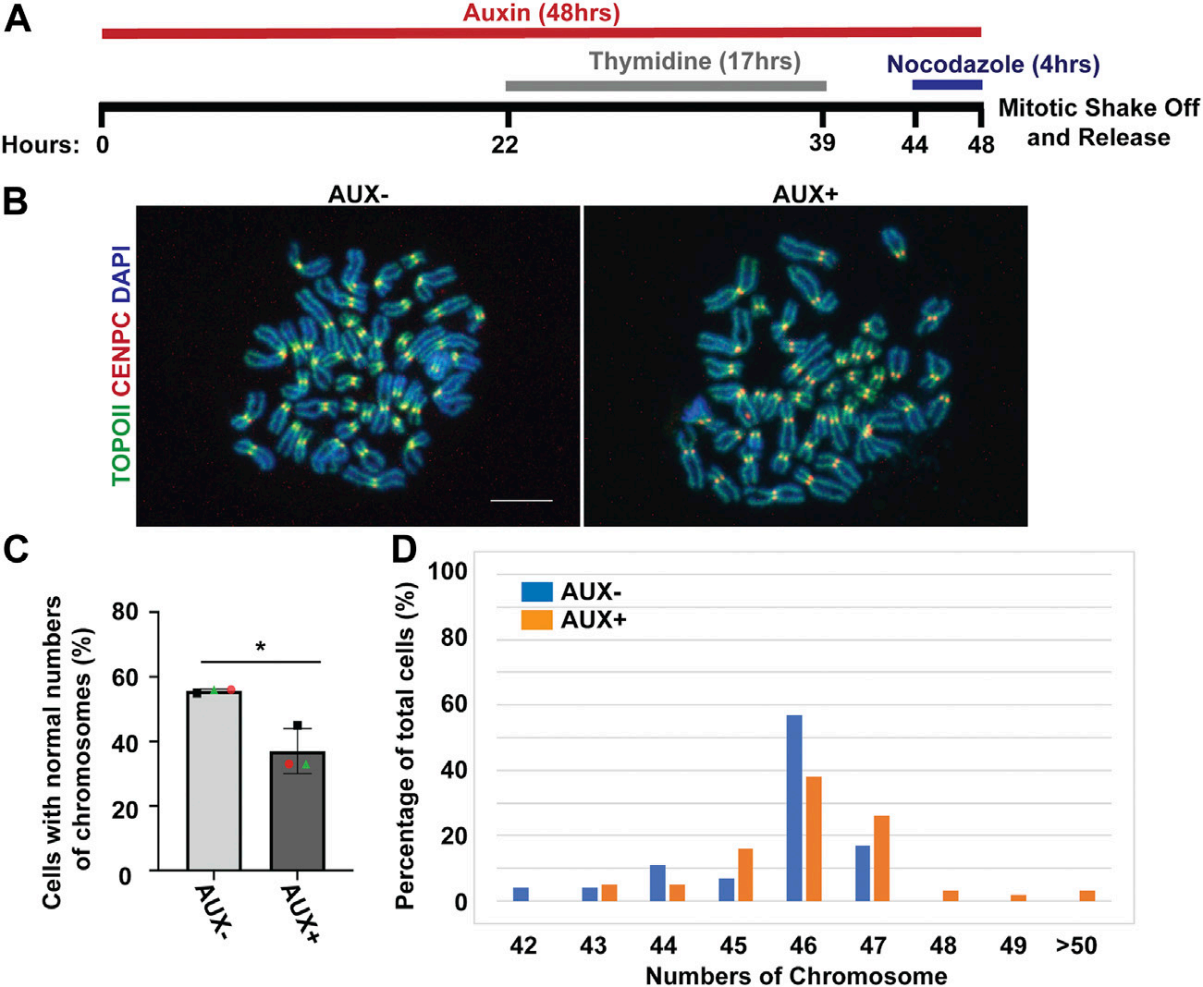
The EWSR1 knockdown promotes the induction of aneuploidy. **(A)**. Schematic diagram for the mitotic synchronization of AID-EWSR1 knockdown (synchronized to mitosis using thymidine/nocodazole, and treated with AUX for 48 h concurrently) cells. The mitotic cells were harvested 30 min after released from the arrest. **(B)**. Representative images of chromosomes visualized with anti-CENP-C (red), and anti-Topoisomerase II (green) obtained from AUX- and AUX + cells. Scale bar = 10 um. **(C)**. The percentage of cells that displayed normal 46 numbers of chromosomes was decreased in AUX + cells compared to AUX-cells. 18-24 cells per sample were counted from *n* = 3 experiments (Total of *n* = 56 cells from AUX-, and of *n* = 62 cells from AUX+). Two-tailed paired t-test, *p < 0.05. **(D)**. The percentage of of cells with each of the chromosome numbers in AUX− and AUX + cells *n* = 3 experiments.

Because the EWSR1 knockdown (AUX+) cells displayed a higher incidence of lagging chromosomes compared to the control (AUX-) cells within a single cell cycle (Figure 2), we further examined whether the AUX + cells result in the induction of aneuploidy after 1 cell division. The cells were treated with Auxin for 48 h combined with cell synchronization because this protocol allowed us to efficiently deplete EWSR1, and then quantify ploidy after one mitosis (Figure 6A). Samples were spread onto slides using a cytospin technique to prepare chromosome spreads, and were subjected to immunocytochemistry using anti-CENP-C (red, a marker for centromere) or anti-TOPO2A (green, a marker to visualize the chromosomes). The representative images of the chromosomes from two sample groups are shown in Figure 6B. When the numbers of chromosomes in both sample groups were scored (43-50 cells per sample, *n* = 3 experiments), the EWSR1 knockdown (AUX+) cells displayed higher incidence of aberrant numbers of chromosomes compared to the control (AUX-) cells (Figures 6C, D; the percentages of the chromosomal number in both sample groups). Together, the results confirm that the EWSR1 reduces the frequency of aneuploidy within a single cell division.

Our previous study showed that EWSR1 interacts with Aurora B, whereas EWSR1:R565A, in which arginine 565 is changed to alanine, displayed a reduced level of the interaction (Park et al., 2016). To evaluate whether EWSR1-Aurora B interaction is required for preventing the induction of aneuploidy, we employed the CRISPR/Cas9 system to generate two new replacement stable cell lines that have an integration of Tet-on *EWSR1-mCherry* (the *mCherry* tag is fused to the C-terminus of *EWSR1*), and *EWSR1:R565A-mCherry* constructs at a safe harbor *AAVS1* locus of the DLD-1 (*AID-EWSR1/AID-EWSR1*) cell line. The CRISPR/Cas9 DNA constructs were transfected into DLD-1 (*AID-EWSR1/AID-EWSR1*) cells, and the colonies were selected with puromycin (Supplementary Figure S6). Next, the presence of mCherry signals in the colonies were visually screened. Among the twenty four colonies, two mCherry positive colonies for both *EWSR1-mCherry* and *EWSR1:R565A-mCherry* transfected cells were isolated, respectively. The cells were treated with 500 µM of Auxin (AUX+), and 1 μg/mL of doxycycline (DOX) for 24 h. The knockdown of EWSR1 and expression of the exogenous EWSR1-mCherry or EWSR1:R565A-mCherry mutant within each of the cell lines were verified with immunocytochemistry, and with western blotting using anti-FLAG and anti-mCherry antibodies obtained from cells (Supplementary Figures S7, S8).

**FIGURE 7:**
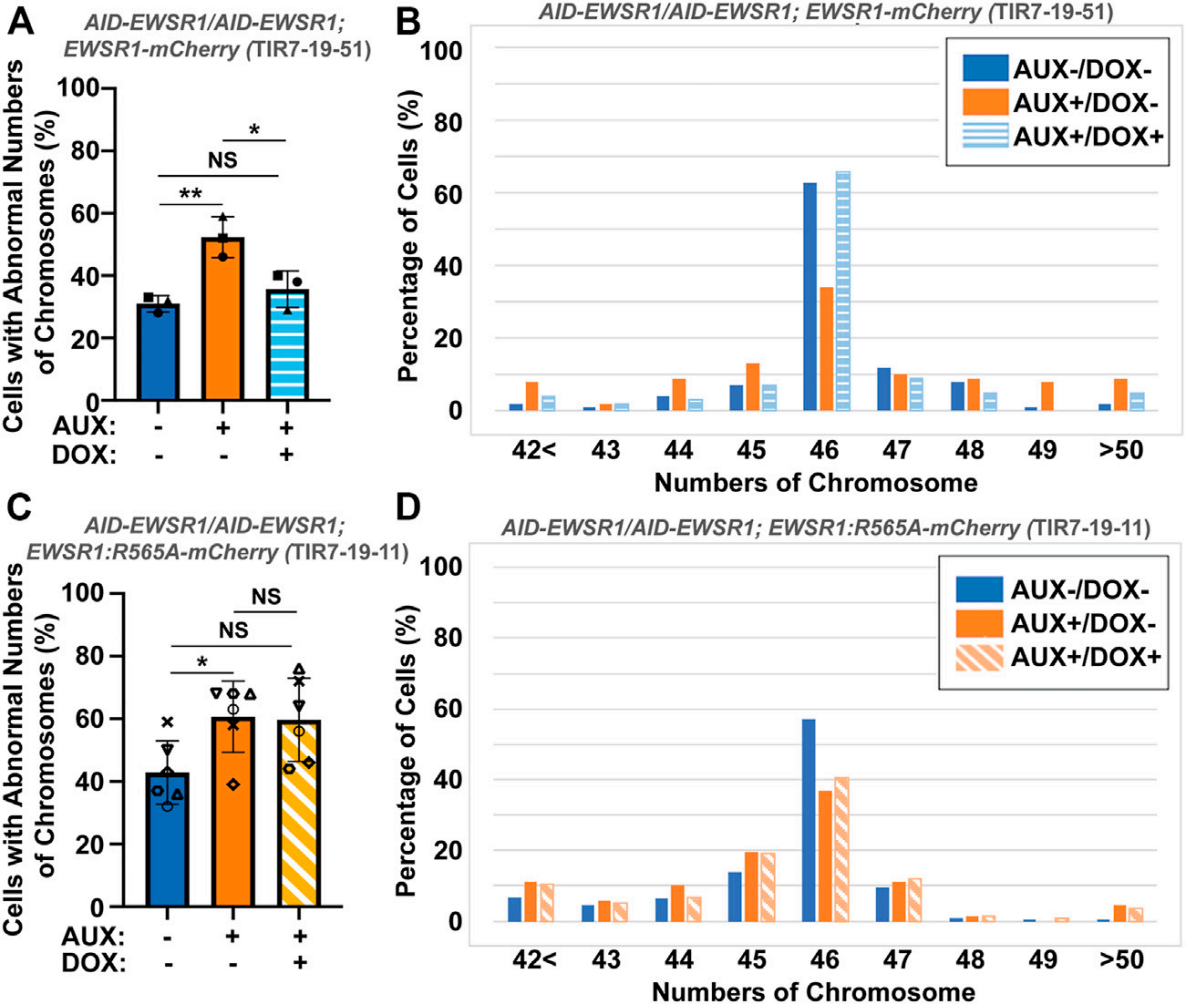
The interaction between EWSR1 and Aurora B prevents the induction of aneuploidy. **(A)**. The high incidence of aberrant chromosome numbers in AUX+/DOX-treated cells was rescued by the expression of EWSR1 overexpression (AUX+/DOX+) cells (28-59 cells per sample, *n* = 3 experiments). One-way ANOVA with Tukey multiple comparison test., **p < 0.01, *p < 0.05. NS = Non-significant. **(B).** The percentages of cells with each chromosome numbers in AUX-/DOX- (*n* = 122), AUX+/DOX- (*n* = 117), and AUX+/DOX+(*n* = 106) cells. **(C)**. The high incidence of aberrant chromosome numbers in AUX+/DOX-cells was not rescued by the expression of EWSR1:R565A in AUX+/DOX + cells. (35-42 cells per sample, *n* = 6 experiments, One-way ANOVA with Tukey multiple comparison test. *p < 0.05, NS = non-significant. **(D)**. The percentage of cells with each chromosome number in AUX-/DOX- (*n* = 221), AUX+/DOX- (*n* = 228), and AUX+/DOX+(*n* = 219) cells.

To evaluate whether the interaction between EWSR1 and Aurora B is required for the reduction of aneuploidy, both (*AID-EWSR1/AID-EWSR1; EWSR1-mCherry*) and (*AID-EWSR1/AID-EWSR1; EWSR1:R565A-mCherry*) DLD-1 cells were treated with combinations of AUX and DOX (AUX-/DOX-, AUX+/DOX-, and AUX+/DOX+) for 48 h, and the chromosomes were spread onto slides using a cytospin technique, followed by immunocytochemistry using anti-TOPO2A and anti-CENP-C. The treatment of the (*AID-EWSR1/AID-EWSR1; EWSR1-mCherry*) cells with AUX induced a high incidence of aneuploidy compared to non-treated (AUX-/DOX-) cells, whereas EWSR1 knockdown combined with induction of EWSR1-mCherry expressing (AUX+/DOX+) cells resulted in a near normal incidence of aneuploidy, confirming that EWSR1-mCherry is functional in reducing aneuploidy (Figure 7A). Percentages of chromosome numbers per cell are listed in Figure 7B. The treatment of the (*AID-EWSR1/AID-EWSR1; EWSR1:R565A-mCherry*) cells with AUX to knock down wild-type EWSR1 consistently induced high incidence of aneuploidy compared to non-treated (AUX-/DOX-) cells, however, the EWSR1 knockdown/EWSR1:R565A-mCherry expressing (AUX+/DOX+) cells did not rescue the high incidence of aneuploidy (Figures 7C, D). These results demonstrate that the EWSR1 allele with reduced interaction with Aurora B is not effective in reducing aneuploidy and suggests, therefore, that the interaction between EWSR1 and Aurora B is required for the prevention of aneuploidy.

## Discussion

**FIGURE 8:**
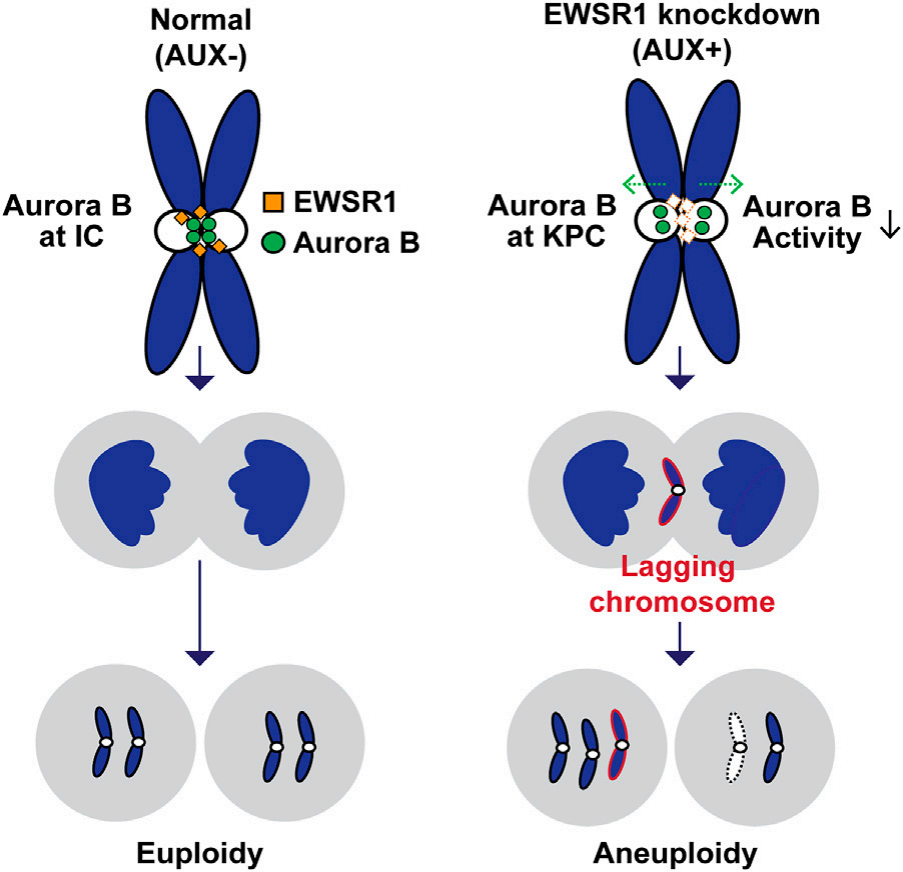
Schematic model of the study. The EWSR1 knockdown leads to the increased incidence of relocation of Aurora B from inner centromere to KPC (prometaphase and metaphase), of lagging chromosomes (anaphase), and of aneuploidy (after a single mitosis). Our data suggests that the EWSR1 prevents the induction of aneuploidy through interaction with Aurora B. Despite of the induction of chromosome mis-segregation in EWSR1 knockdown cells, the cells do not arrest at mitosis, and it is likely that the cells are overriding the error collection process.

The aim of this study was to determine if depletion of EWSR1 protein in human cells causes aneuploidy and, if so, to elucidate the molecular function of EWSR1 in preventing the induction of aneuploidy. Here, we demonstrate that the conditional knockdown of EWSR1 in DLD-1 cells for 1 cell cycle reduces Aurora B at the inner centromere and to enrichs the protein at the KPC during pro/metaphase, increases the incidence of lagging chromosomes during anaphase, and induces a high incidence of aneuploidy after one mitosis. Note that this is the first study to show the EWSR1 knockdown dependent aneuploidy induction in human cells. Importantly, we propose that EWSR1 inhibits the induction of aneuploidy by interacting with Aurora B. Despite that the EWSR1 knockdown cells induced lagging chromosomes and aneuploidy during mitosis, the cells failed to undergo mitotic arrest. Our study highlights a potential new role of EWSR1 in facilitating Aurora B resulted in a near normal error correction during mitosis, and cells lacking EWSR1 may induce chromosomal instability (CIN) by overriding the process of error correction (Figure 8).

Future studies are required to elucidate how EWSR1 prevents the induction of lagging chromosomes and aneuploidy, and how EWSR1 knockdown cells escape from mitotic arrest. In general, microtubule turnover occurs until it establishes a proper bi-oriented microtubule-kinetochore attachment during prometaphase. When there is an attachment error, Aurora B is directly recruited to the kinetochore, where it corrects the error by increasing the frequency of microtubule detachment (Caldas et al., 2013; Broad et al., 2020). Our data show that the EWSR1 knockdown promotes the re-location of Aurora B to the kinetochore, and the reduction of phosphorylation of the Histone H3 Ser28 that is required for the chromosomal condensation during early mitosis (Figure 3 and Supplementary Figure S4). Therefore, the data suggest that EWSR1 prevents the induction of error or its correction through the regulation of the kinase activity of Aurora B in normal cell. Other possibility for the function of EWSR1 is that the protein promotes detachment of microtubules by regulating the kinase activity of Aurora B at the erroneously attached kinetochore. For example, EWSR1 may be required for the Aurora B dependent phosphorylation of its substrates, MCAK and Kif2b (microtubule depolymerizing proteins) because both proteins inhibit the induction of chromosome mis-segregation by promoting the microtubule turnover (Wordeman et al., 2007). Another possibility is that EWSR1 facilitates the Aurora B dependent phosphorylation of Hec1/Ndc80, a core element of kinetochores that activates the detachment of microtubules from kinetochores and promotes polymerization of the microtubule at its plus end (DeLuca et al., 2006). Another potential mechanism required for faithful chromosome segregation is that EWSR1 regulates R-loops at centromeres and resolves lagging chromosome by activating the ATR-Aurora B axis (Kabeche et al., 2018). One attractive model is that EWSR1 functions as an adapter molecule to recruit molecules listed above, and to coordinate the two pathways to resolve the error to prevent the induction of lagging chromosomes. Lagging chromosomes are often induced by merotelic attachment, a condition where a kinetochore is attached to microtubules nucleated from two opposite centrosomes (Cimini et al., 2001a). It is noteworthy that our assay system to measure the lagging chromosomes employed the mitotic synchronization protocol using thymidine and nocodazole. The nocodazole induces higher incidence of merotelic attachment, thus the nocodazole treatment in the EWSR1 cells may have enabled us to observe the pronounced activity of EWSR1, in preventing the induction of lagging chromosome (Cimini et al., 2001a).

In addition to *EWSR1-FLI1* expression in Ewing sarcoma, the *EWSR1* gene fuses to various genes in other cancers (e.g., *EWSR1/ELF5* in acute myeloid leukemia, *EWSR1/NR4A3* in extraskeletal myxoid chondrosarcomas, and *EWSR1/CHOP* in liposarcoma etc) (Panagopoulos et al., 2002; Matsui et al., 2006; Filion et al., 2009; Endo et al., 2016; Panza et al., 2021). As a result of the formation of these *EWSR1*-fusion genes, the cells share a common genetics alteration; loss of one wildtype *EWSR1* allele. If the haploinsufficiency of EWSR1 contributes to the pathogenesis of these diseases, an approach involving modulation of the level of EWSR1 may be an effective approach to treating *EWSR1*-fusion expressing cancer patients. For this reason, it is essential to investigate the activity of loss of *EWSR1* allele during tumorigenesis. Although, the effect of haploinsufficiency of EWSR1 is under-studied, multiple studies indicated the importance of this concept. Our previous study using zebrafish demonstrated that the loss of one *ewsa* (a homologue of human *EWSR1*) allele (*ewsa*/*wt*) promotes tumorigenesis in the *tp53* mutation background (Park et al., 2016). In addition, one study demonstrated that the expression levels of EWSR1 proteins in Ewing sarcoma cell lines are lower than the cell lines that do not express the EWSR1-fusion gene (Huang et al., 2012). The study demonstrated that the knockdown of EWSR1 in the Ewing sarcoma cells facilitates the interaction between the EWSR1 proteins and the 3’ untranslated region of PRAS40 (Akt substrate) mRNA, ultimately leading to the increased levels of PRAS40 protein. The study demonstrated that the haploinsufficiency of the EWSR1 drives the Ewing sarcoma development (Huang et al., 2012). The unique (*AID-EWSR1*/*AID-EWSR1*) homozygous cell line established in this study enables the conditional knockdown of EWSR1, and to study its effect after a single cell cycle. Thus, the system permitting us to study the primary defects caused by the EWSR1 knockdown minimizing additional secondary defects. Future study will require the establishment of (*AID-EWSR1*/*wt*) heterozygous cell line, and to elucidate the effect of EWSR1 happloinsufficiency derived from one *EWSR1* allele. Furthermore, such cell lines will enable us to investigate the interplay between the happloinsufficiency of EWSR1 and the *EWSR1*-fusion in a systematic manner. The cell line that enables the conditional knockdown and expression is a powerful tool to investigate the multiple genotypes and their phenotypes. Our study provides a unique tool for clarifying role of EWSR1 in maintaining the chromosomal stability by preventing the induction of lagging chromosomes and aneuploidy through regulation of Aurora B.

## Data Availability

The original contributions presented in the study are included in the article/Supplementary Material, further inquiries can be directed to the corresponding author.
