# Supplementary figures and images for: EWSR1 prevents the induction of aneuploidy through direct regulation of Aurora B
Source: Front Cell Dev Biol. 2023 Feb 15;11:987153. doi: 10.3389/fcell.2023.987153 (PMC9975954; doi:10.3389/fcell.2023.987153)

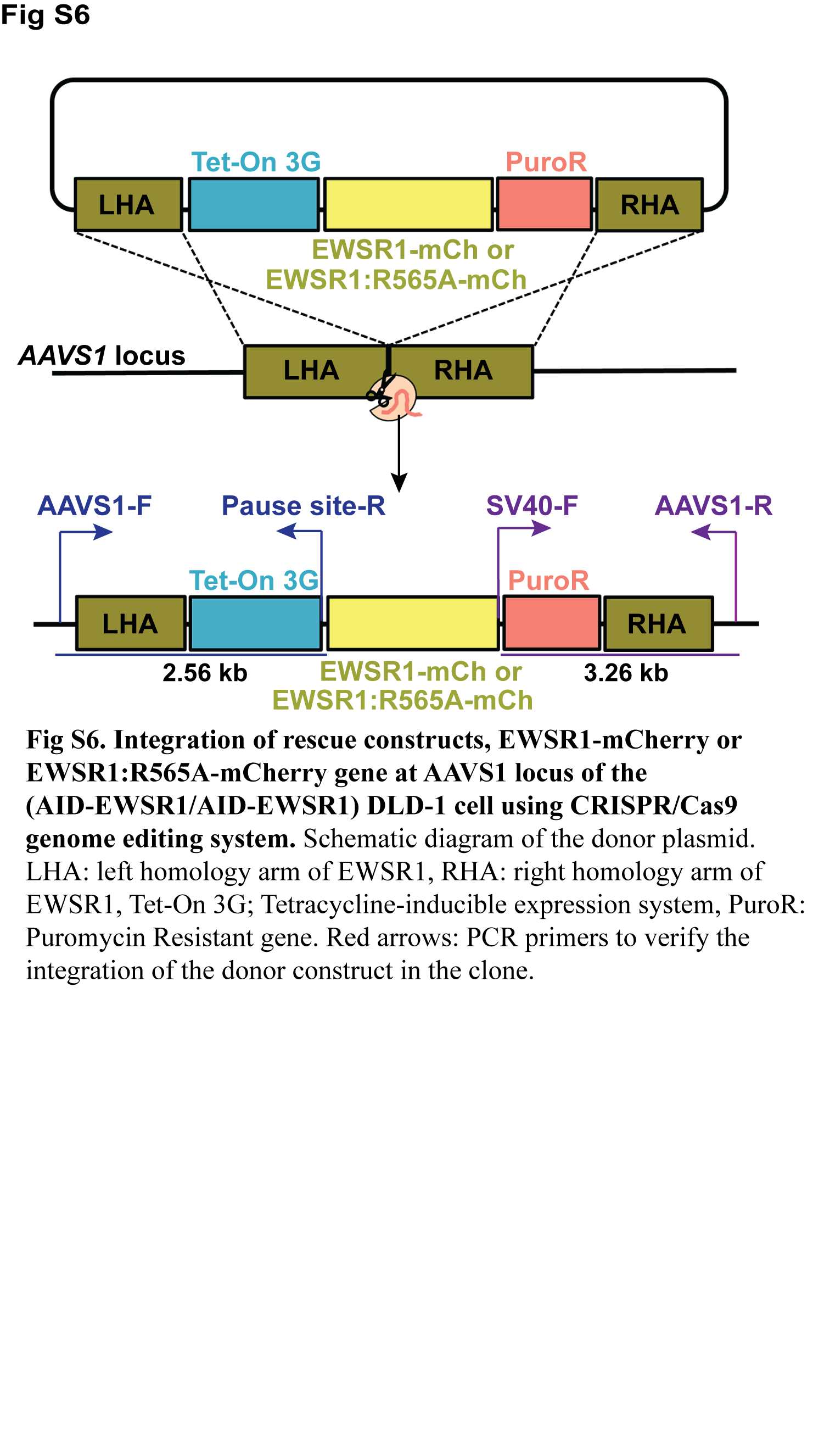

Supplement: Supplementary file 1 [file Image6.tif]

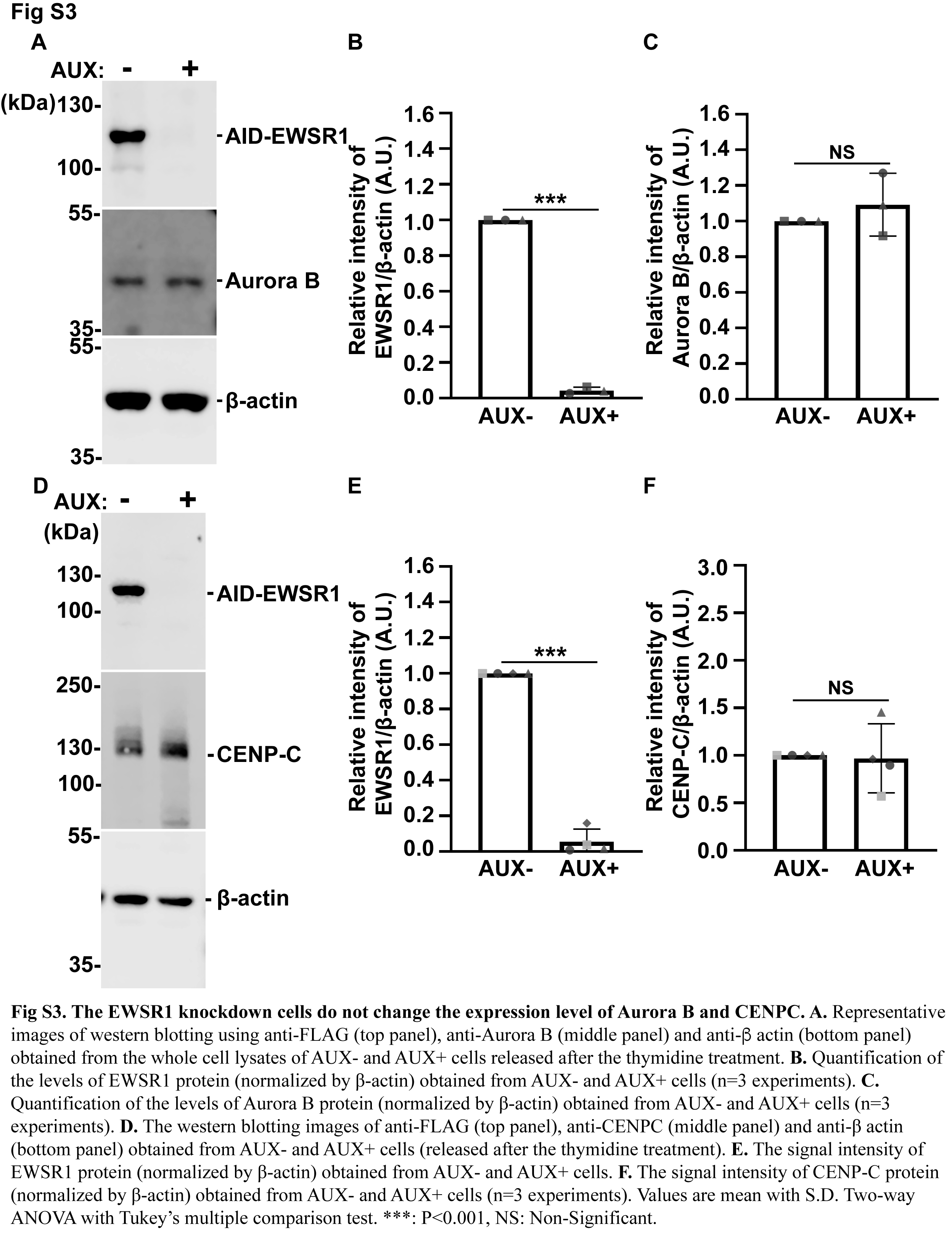

Supplement: Supplementary file 2 [file Image3.tif]

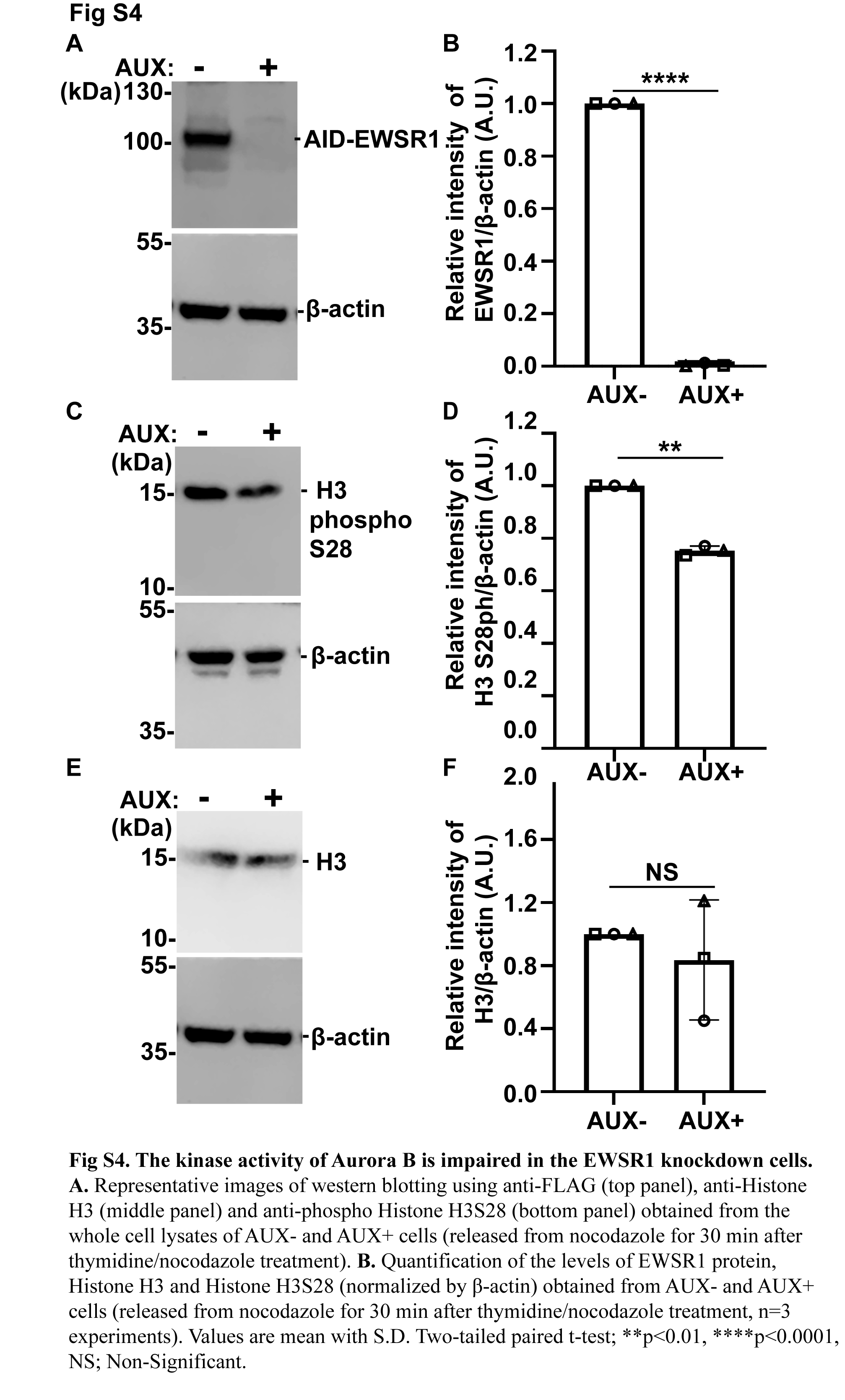

Supplement: Supplementary file 3 [file Image4.tif]

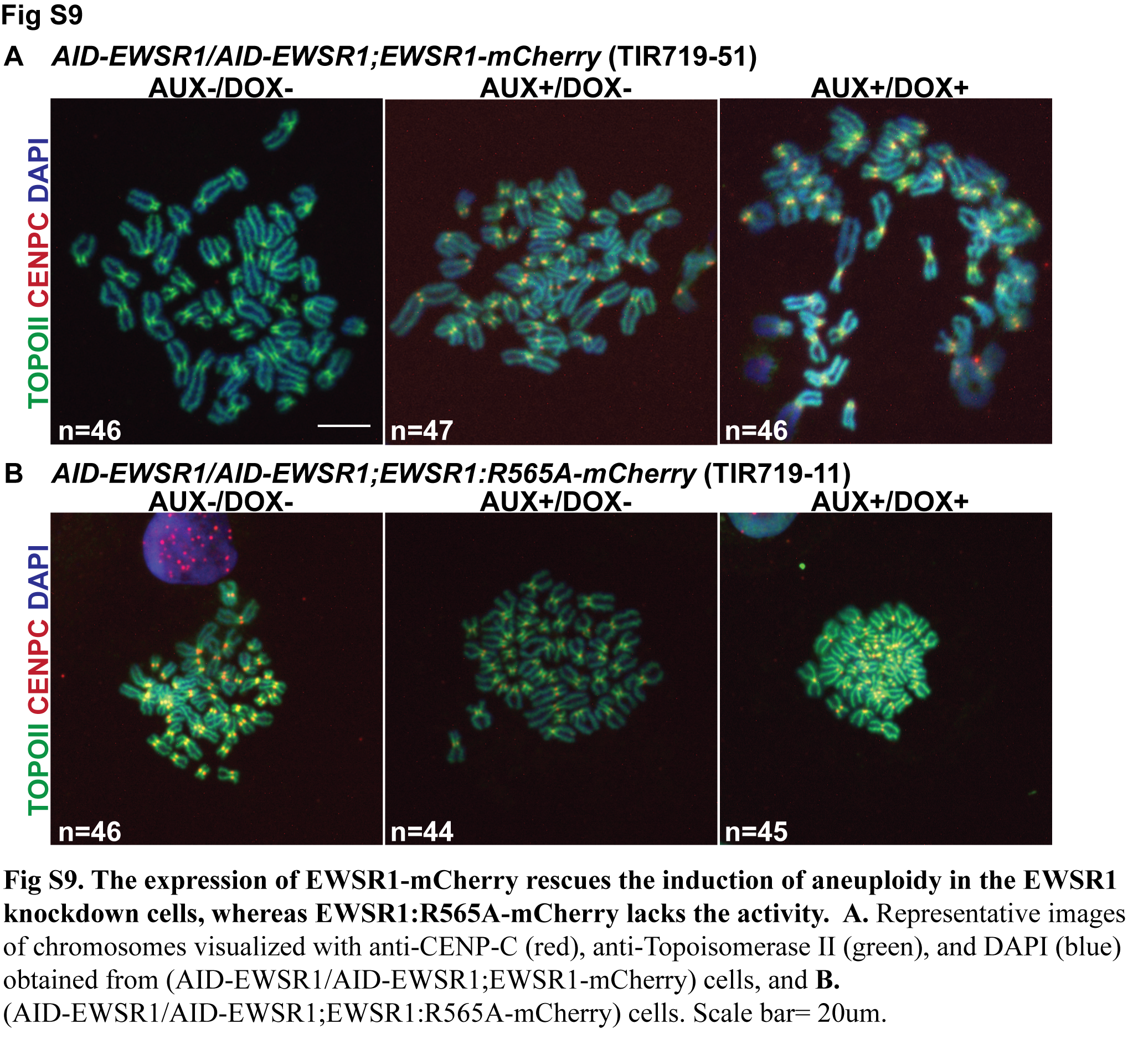

Supplement: Supplementary file 4 [file Image9.tif]

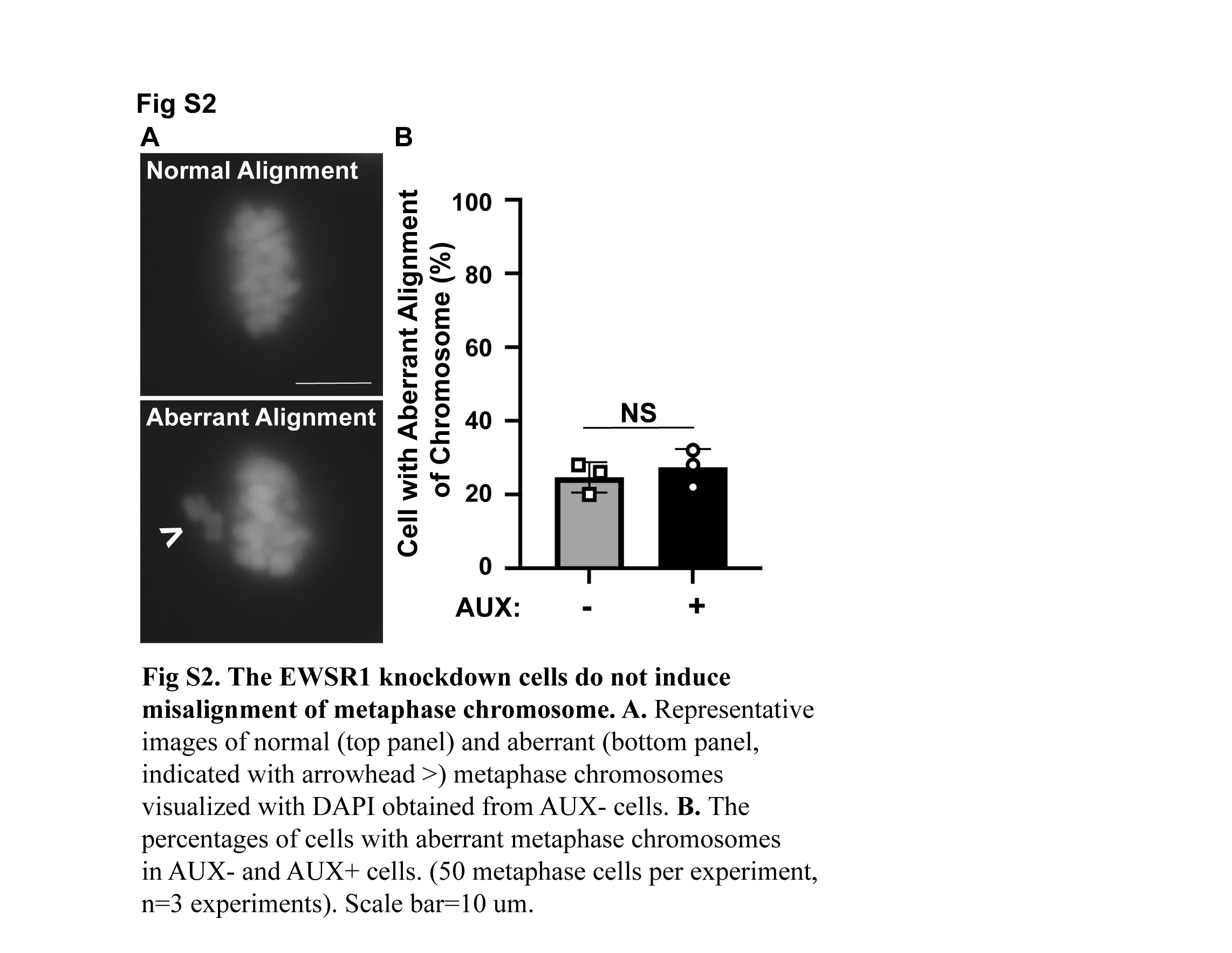

Supplement: Supplementary file 5 [file Image2.tif]

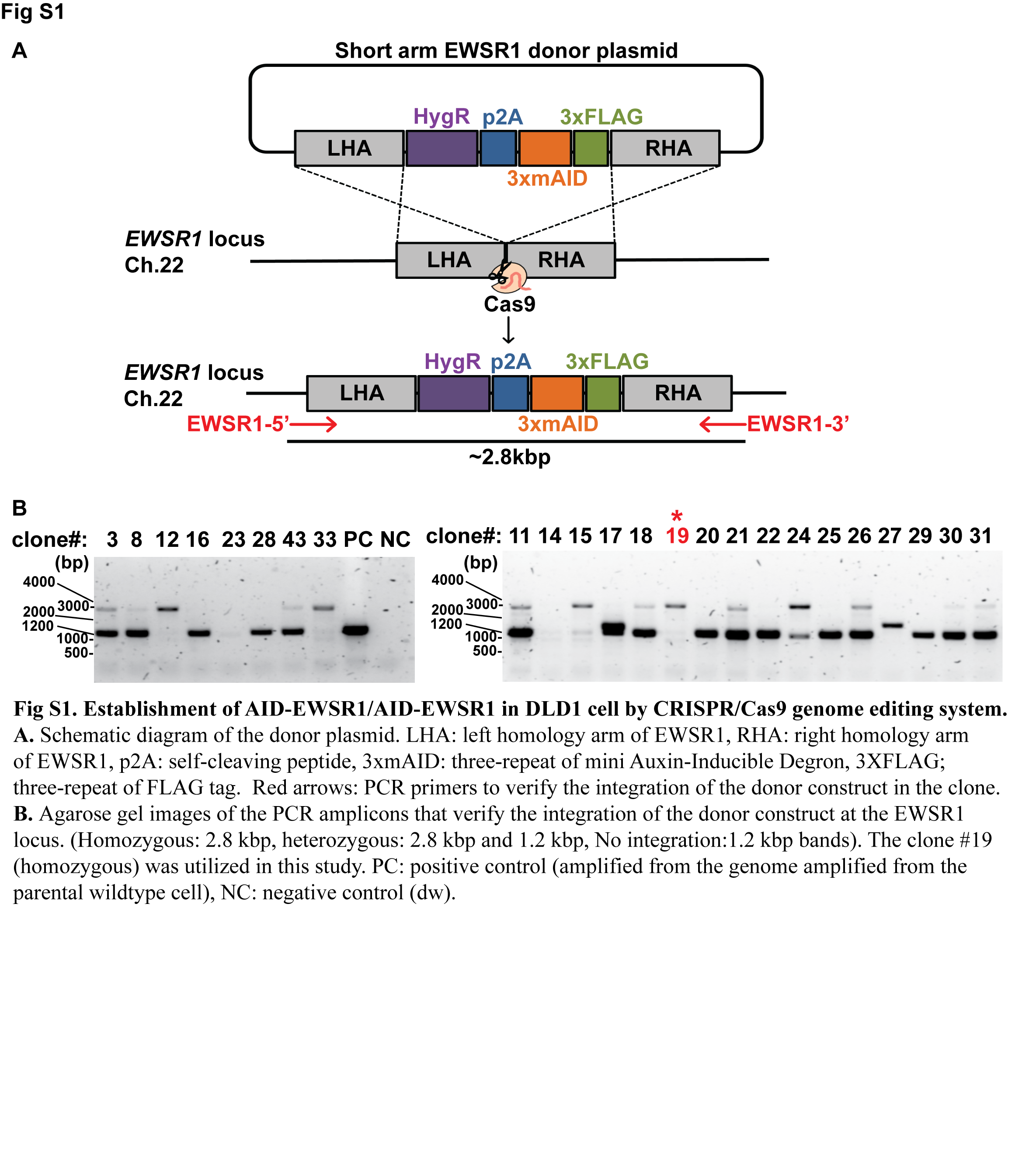

Supplement: Supplementary file 6 [file Image1.tif]

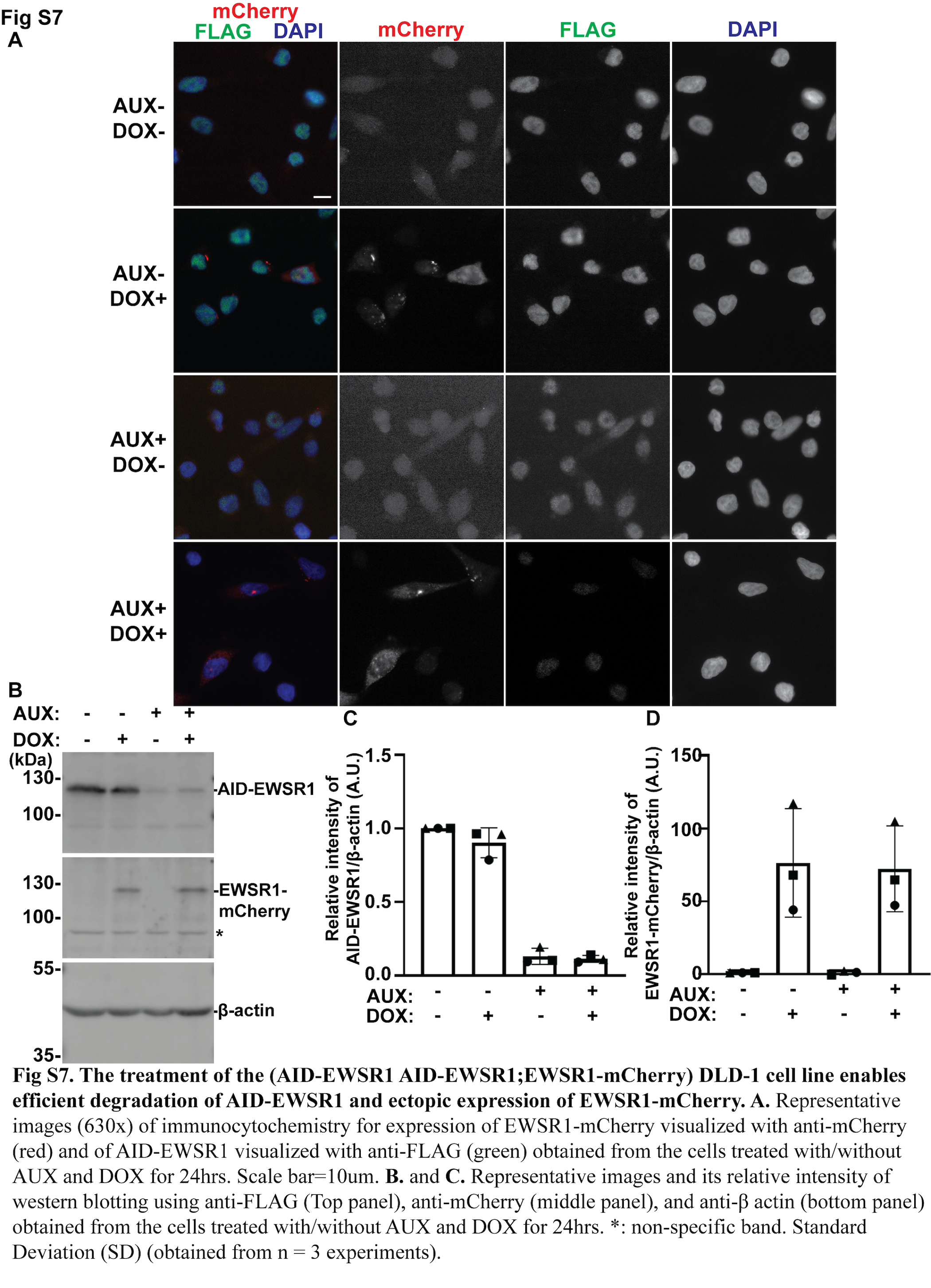

Supplement: Supplementary file 7 [file Image7.tif]

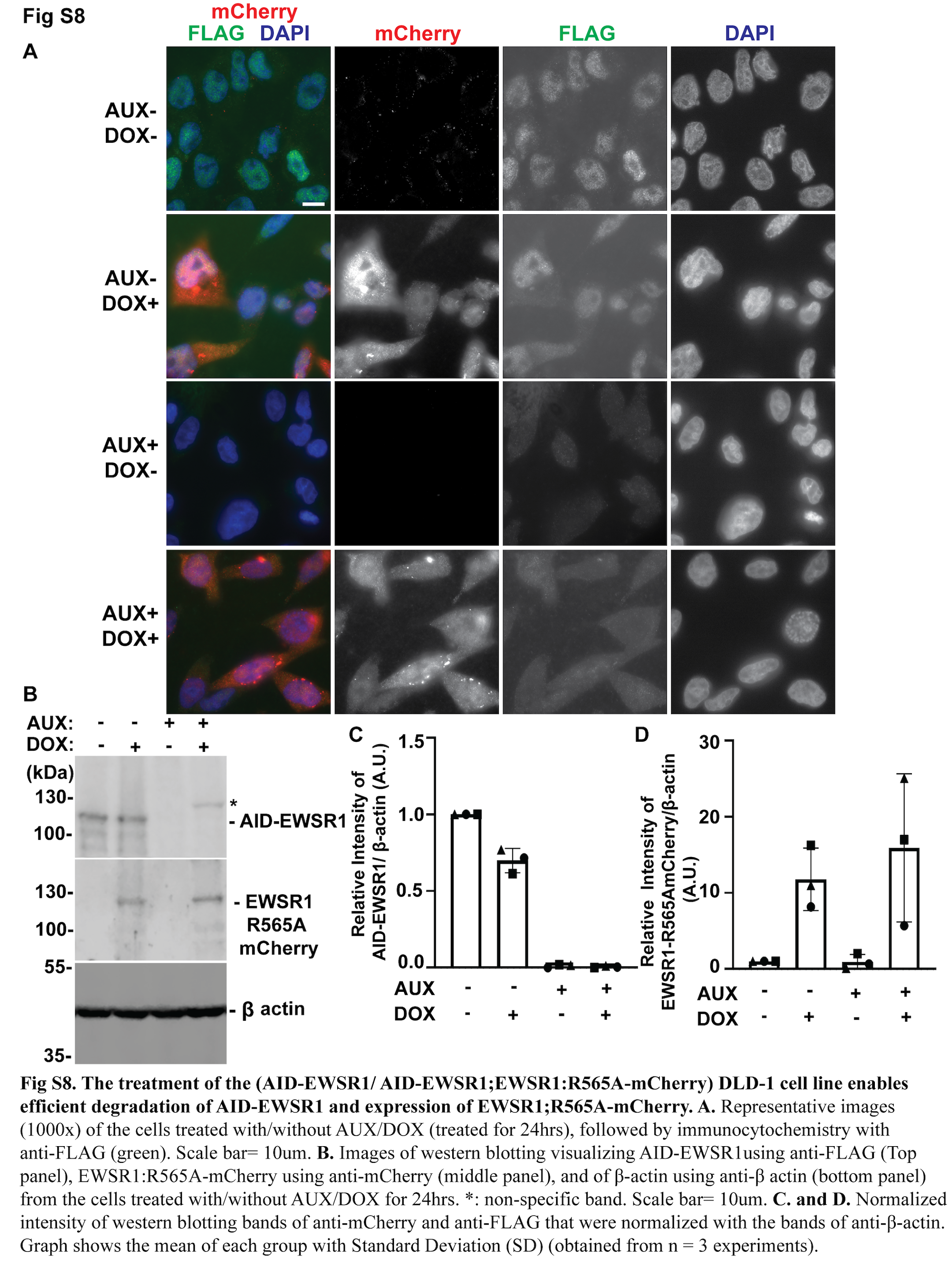

Supplement: Supplementary file 9 [file Image8.tif]

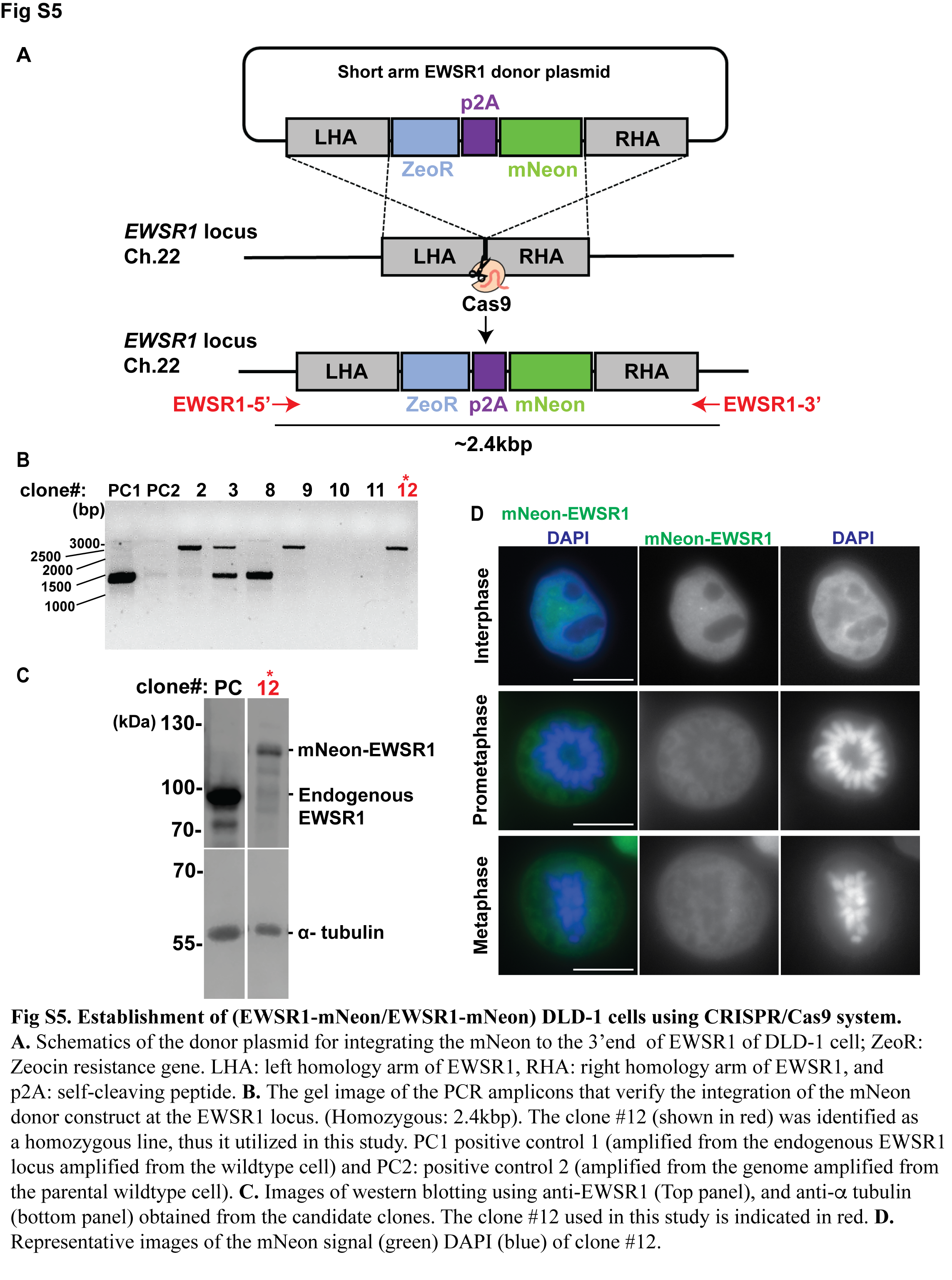

Supplement: Supplementary file 10 [file Image5.tif]
