# Supplementary material for: EWSR1 prevents the induction of aneuploidy through direct regulation of Aurora B
Source: Front Cell Dev Biol. 2023 Feb 15;11:987153. doi: 10.3389/fcell.2023.987153 (PMC9975954; doi:10.3389/fcell.2023.987153)
Supplement: Supplementary file 8 [file DataSheet1.PDF]

Title:
EWSR1 prevents the induction of aneuploidy by regulating the localization of Aurora B at inner centromere

Supplementary Materials

Fig S1. Establishment of *AID-EWSR1/AID-EWSR1* in DLD1 cell by CRISPR/Cas9 genome editing system.

A. Schematic diagram of the donor plasmid. LHA: left homology arm of *EWSR1*, RHA: right homology arm of *EWSR1*, p2A: self-cleaving peptide, 3xmAID: three-repeat of mini Auxin-Inducible Degron, 3XFLAG; three-repeat of FLAG tag. Red arrows: PCR primers to verify the integration of the donor construct in the clone. **B**. Agarose gel images of the PCR amplicons that verify the integration of the donor construct at the *EWSR1* locus. (Homozygous: 2.8kbp, heterozygous: 2.8kbp and 1.2kbp, No integration:1.2 kbp bands). The clone #19 (homozygous) was utilized in this study. PC: positive control (amplified from the genome amplified from the parental wildtype cell), NC: negative control (dw).

Fig S2. The EWSR1 knockdown cells do not induce misalignment of metaphase chromosome.

A. Representative images of normal (top panel) and aberrant (bottom panel, indicated with arrowhead >) metaphase chromosomes visualized with DAPI obtained from AUX- cells. **B.** The percentages of cells with aberrant metaphase chromosomes in AUX- and AUX+ cells. (50 chromosomes per experiment, n=3 experiments). Scale bar=10 um.

Fig S3. The EWSR1 knockdown cells do not change the expression level of Aurora B and CENPC.

A. Representative images of western blotting using anti-FLAG (top panel), anti-Aurora B (middle panel) and anti-β actin (bottom panel) obtained from the whole cell lysates of AUX- and AUX+ cells released after the thymidine treatment. **B.** Quantification of the levels of EWSR1 protein (normalized by β-actin) obtained from AUX- and AUX+ cells (n=3 experiments). **C.** Quantification of the levels of Aurora B protein (normalized by β-actin) obtained from AUX- and AUX+ cells (n=3 experiments). **D.** The western blotting images of anti-FLAG (top panel), anti-CENPC (middle panel) and anti-β actin (bottom panel) obtained from AUX- and AUX+ cells (released after the thymidine treatment). **E.** The signal intensity of EWSR1 protein (normalized by β-actin) obtained from AUX- and AUX+ cells. **F.** The signal intensity of CENP-C protein (normalized by β-actin) obtained from AUX- and AUX+ cells (n=3 experiments). Values are mean with S.D. Two-way ANOVA with Tukey's multiple comparison test. ***: $P<0.001$, NS: Non-Significant.

Fig S4. The kinase activity of Aurora B is impaired in the EWSR1 knockdown cells.

A. Representative images of western blotting using anti-FLAG (top panel), anti-Histone H3 (middle panel) and anti-phospho Histone H3S28 (bottom panel) obtained from the whole cell lysates of AUX- and AUX+ cells (released from nocodazole for 30 min after thymidine/nocodazole treatment). **B.** Quantification of the levels of EWSR1 protein, Histone H3 and Histone H3S28 (normalized by β-actin) obtained from AUX- and AUX+ cells (released from nocodazole for 30 min after thymidine/nocodazole treatment, n=3 experiments). Values are mean with S.D. Two-tailed paired t-test; **p<0.01, ****p<0.0001, NS; Non-Significant.

Fig S5. Establishment of (*EWSR1-mNeon/EWSR1-mNeon*) DLD-1 cells using CRISPR/Cas9 system.

A. Schematics of the donor plasmid for integrating the *mNeon* to the 3'end of *EWSR1* of DLD-1 cell; ZeoR: Zeocin resistance gene. LHA: left homology arm of *EWSR1*, RHA: right homology arm of *EWSR1*, and p2A: self-cleaving peptide. **B**. The gel image of the PCR amplicons that verify the integration of the mNeon donor construct at the *EWSR1* locus. (Homozygous: 2.4kbp). The clone #12 (shown in red) was identified as a homozygous line, thus it utilized in this study. PC1 positive control 1 (amplified from the endogenous EWSR1 locus amplified from the wildtype cell) and PC2: positive contro 2 (amplified from the genome amplified from the parental wildtype cell). **C.** Images of western blotting using anti-EWSR1 (Top panel), and anti-α tubulin (bottom panel) obtained from the candidate clones. The clone #12 used in this study is indicated in red. **D.** Representative images of the mNeon signal (green) DAPI (blue) of clone #12.

Fig S6. Integration of rescue constructs, *EWSR1-mCherry* or *EWSR1:R565A-mCherry* gene at *AAVS1* locus of the (*AID-EWSR1/AID-EWSR1*) DLD-1 cell using CRISPR/Cas9 genome editing system.

A. Schematic diagram of the donor plasmid. LHA: left homology arm of *EWSR1*, RHA: right homology arm of *EWSR1*, Tet-On 3G; Tetracycline-inducible expression system, PuroR: *Puromycin Resistant* gene. Red arrows: PCR primers to verify the integration of the donor construct in the clone.

Fig S7. The treatment of the (*AID-EWSR1 AID-EWSR1;EWSR1-mCherry)* DLD-1 cell line enables efficient degradation of AID-EWSR1 and ectopic expression of EWSR1-mCherry.

A. Representative images of immunocytochemistry for expression of EWSR1-mCherry visualized with anti-mCherry (red) and of AID-EWSR1 visualized with anti-FLAG (green) obtained from the cells treated with/without AUX/DOX (AUX-/DOX-, AUX-/DOX+, AUX+/DOX- and AUX+/DOX+) for 24hrs. Merged images (left panel), anti-mCherry (second from left panel), anti-FLAG (second from right panel), and DAPI (right panel). Scale bar= 10um. **B.** Representative images of western blotting using anti-FLAG (Top panel), anti-mCherry (middle panel), and anti-

β actin (bottom panel) obtained from the cells with AUX-/DOX-, AUX-/DOX+, AUX+/DOX- and AUX+/DOX+ (treated for 24hrs). *: non-specific band. **C.** Relative intensity of the bands of anti-mCherry (normalized to bands of anti-β-actin) obtained from western blotting. **D.** Relative intensity of the bands of anti-FLAG (normalized to bands of anti-β-actin) obtained from western blotting. Graph shows the mean of each group with Standard Deviation (SD) (obtained from n = 3 experiments).

Fig S8. The treatment of the (*AID-EWSR1/ AID-EWSR1;EWSR1:R565A-mCherry)* DLD-1 cell line enables efficient degradation of AID-EWSR1 and expression of EWSR1;R565A-mCherry.

A. Representative images of the cells treated with/without AUX/DOX (treated for 24hrs), followed by the visualization of the expression of EWSR1:R565A-mCherry with anti-mCherry (red) and of AID-EWSR1 visualized with anti-FLAG (green) by immunocytochemistry. Merged images (left panel), anti-mCherry (second from left panel), anti-FLAG (second from right panel), and DAPI (right panel). Scale bar= 10um. **B.** Images of western blotting visualizing AID-EWSR1using anti-FLAG (Top panel), EWSR1:R565A-mCherry using anti-mCherry (middle panel), and of β-actin using anti-β actin (bottom panel) from the cells with AUX-/DOX-, AUX-/DOX+, AUX+/DOX- and AUX+/DOX+ treated for 24hrs. *: non-specific band. **C.** Normalized intensity of western blotting bands of anti-mCherry that were normalized with the bands of anti-β-actin. **D.** Normalized intensity of western blotting bands of anti-FLAG, normalized to bands obtained from the usage of anti-β-actin. Graph shows the mean of each group with Standard Deviation (SD) (obtained from n = 3 experiments).

Fig S9. The expression of EWSR1-mCheery rescues the induction of aneuploidy in the EWSR1 knockdown cells, whereas EWSR1:R565A-mCheery lacks the activity.

Representative images of chromosomes visualized with anti-CENPC (red), anti-Topoisomerase II (green), and DAPI (blue) obtained from (*AID-EWSR1/AID-EWSR1;EWSR1-mCherry*) cells. **A**. and (*AID-EWSR1/AID-EWSR1;EWSR1:R565A-mCherry*) cells. **B**. Scale bar= 20um.
